# Seasonal patterns and host traits as drivers of ectoparasite abundance in 3 bat species (*Miniopterus schreibersii*, *Rhinolophus euryale*, and *Rhinolophus ferrumequinum*) in the Central Balkans

**DOI:** 10.1093/cz/zoaf079

**Published:** 2025-12-22

**Authors:** Jelena Burazerović, Sara Arsenijević, Marija Jovanović, Marko Obradović, Snežana Tomanović

**Affiliations:** Faculty of Biology, University of Belgrade, Studentski trg 16, 11000 Belgrade, Serbia; Faculty of Biology, University of Belgrade, Studentski trg 16, 11000 Belgrade, Serbia; Organisation for Respect and Care of Animals—ORCA, Vojislava Ilica 94, 11000 Belgrade, Serbia; Faculty of Mathematics, University of Belgrade, Studentski trg 16, 11000 Belgrade, Serbia; Institute for Medical Research, National Institute of Republic of Serbia, University of Belgrade, Bulevar oslobođenja 18, 11000 Belgrade, Serbia

**Keywords:** bat flies, host traits, host-parasite interactions, mites, seasonal effects, ticks

## Abstract

Ectoparasite-host interactions are influenced by a complex interplay of factors, including host species, body condition, sex, age, reproductive status, and seasonal variations. These interactions are often complex, varying not only between species but also across different biogeographical regions. This study evaluates how seasonal changes and host characteristics affect ectoparasite abundance on 3 bat species, with ectoparasite abundance as the response variable and seasonality and host traits (sex, age, reproductive status, and body condition) as predictor variables. We examined the total ectoparasite burden and the most prevalent ectoparasite species across 3 bat species from 29 localities in the Central Balkans. The abundance of ectoparasites was influenced by season (*Rhinolophus euryale*), host sex (*R. ferrumequinum*), and a combination of season and host sex (*Miniopterus schreibersii*). Overall, the highest ectoparasite numbers were recorded in spring and summer when bat hosts, particularly females and juveniles, form dense colonies, providing optimal conditions for parasite transmission and reproduction. While there were slight variations between species, ectoparasite reproduction generally synchronized with the host's reproductive cycle. In *M. schreibersii*, host pregnancy was associated with increased overall ectoparasite abundance, particularly elevating the numbers of *Ixodes simplex* and *Nycteribia schmidlii*, whereas lactation specifically led to a significant rise in *I. simplex* abundance. Additionally, besides season and host sex, host body condition emerged as a significant trait affecting ectoparasite abundance in *M. schreibersii* and *R. euryale*. This study highlights key regional and species-specific factors shaping bat ectoparasite communities in the Central Balkans, providing a foundation for future research and conservation.

## Graphical Abstract

**Graphical Abstract:**
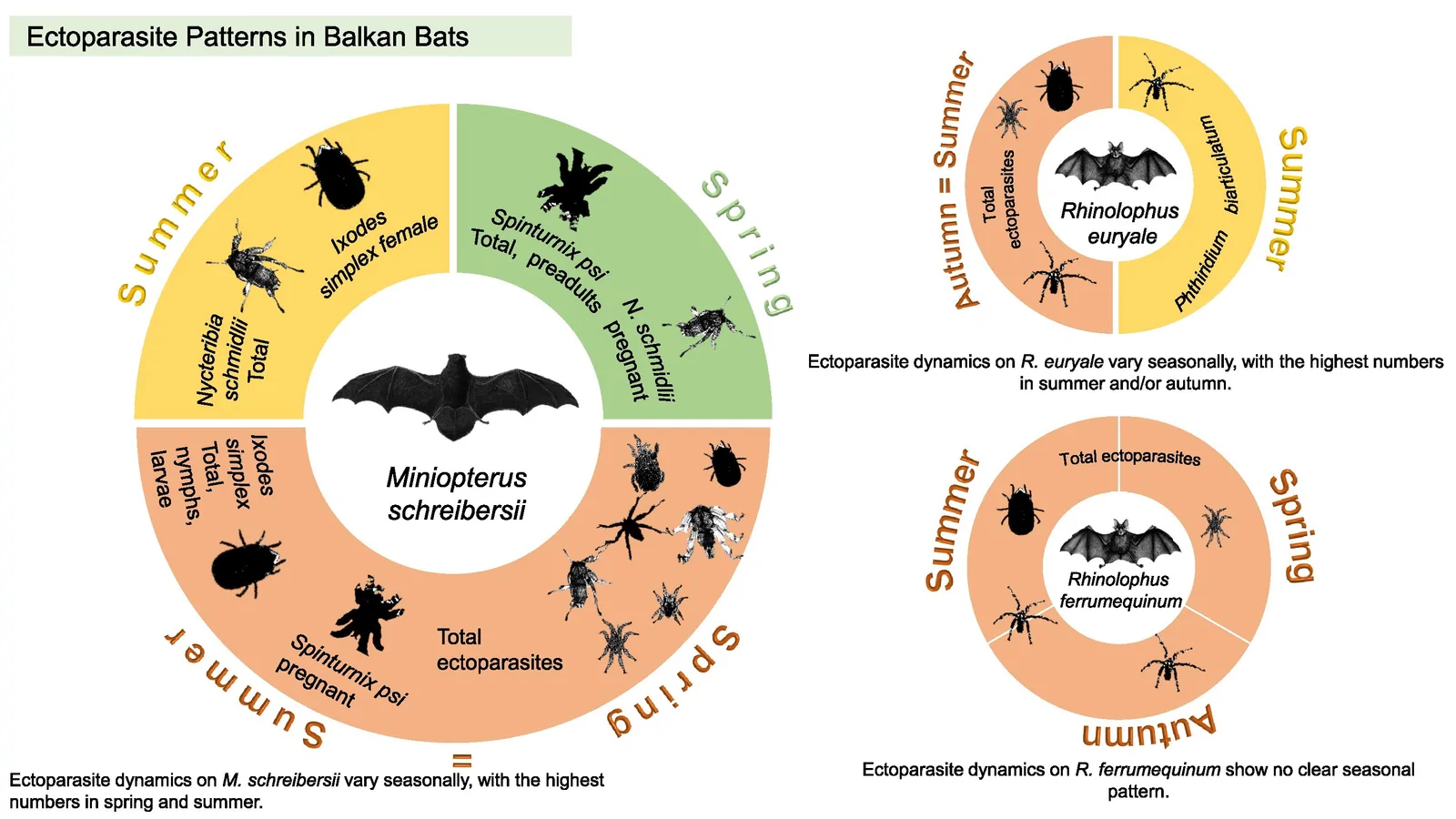

Bats (order Chiroptera) represent one of the most diverse and ecologically significant mammalian groups, playing essential roles in ecosystems, particularly as insect regulators (Kunz et al. 2011). Bats serve as important hosts for various ectoparasites, including bat flies, mites, and ticks, which are known vectors of pathogens impacting both bat and human health (Hornok et al. 2019; Sándor et al. 2021; Corduneanu et al. 2023; Sándor et al. 2024). Notably, these ectoparasites have been associated with bacterial pathogens such as *Bartonella rousetti*, *Candidatus Mycoplasma haemohominis, Rickettsia raoultii*, *Anaplasma phagocytophilum*, and *Neoehrlichia mikurensis*, as well as viral agents like the *Lloviu* virus (Szentiványi et al. 2024). Studying bat ectoparasites provides important insights into host-parasite coevolution, ecological and evolutionary dynamics, wildlife conservation and potential zoonotic disease transmission (Brook and Dobson 2015; Szentiványi et al. 2024). Host-parasite interactions are influenced by host ecology and behavior, which shape predictable patterns in parasite prevalence and community dynamics. However, despite ecological and epidemiological implications, many aspects of bat ectoparasitism remain poorly understood.

Ectoparasite-host interactions are influenced by season and various host-related factors, including species, body condition, sex, age, and reproductive status (Giorgi et al. 2004; Lourenço and Palmeirim 2008; Tai et al. 2022). For instance, the reproductive cycles of 4 bat ectoparasite species (*Nycteribia schmidlii*, *Penicillidia conspicua*, *Spinturnix psi*, and *Ixodes simplex*) parasitizing *Miniopterus schreibersii* in Portugal intensified during the host's pregnancy and nursing periods, particularly affecting pregnant and juvenile bats (Lourenço and Palmeirim 2008). Host body condition has been shown to play a significant role, with previous studies reporting variable correlations. For example, a study on *Rousettus madagascariensis* found a weak positive correlation between host body condition and nycteribiid abundance, suggesting that healthier bats may provide more resources for parasites (Rajemison et al. 2017). Conversely, a negative correlation was reported between bat condition and mite (*S. psi*) load during the nursing season across male, female, and juvenile hosts (*M. schreibersii*) in Portugal (Lourenço and Palmeirim 2007). These examples illustrate that host-specific traits significantly shape ectoparasite infestation patterns.

Seasonality significantly affects ectoparasite dynamics; parasitized bats and their ectoparasitic flies were more abundant during the wet season in the Amazonian savannahs, correlating with increased bat activity and aggregation, which facilitate higher ectoparasite transmission rates (Mejía et al. 2023). Similarly, in Eastern Europe, the prevalence and intensity of *I. simplex* on *M. schreibersii* peaked during spring and summer, coinciding with the formation of maternity colonies (Sándor et al. 2019). These examples underscore the importance of seasonal factors when studying ectoparasite-host interactions.

The Central Balkans hosts a rich diversity of bat species, with Serbia alone recording 31 species, including several of conservation concern such as *M. schreibersii* (Cistrone et al. 2023). These bats play crucial ecological roles as insectivores, contributing to pest control and ecosystem balance (Kunz et al. 2011). Previous studies on bat ectoparasites in the Central Balkans primarily focused on identifying ectoparasite species and analyzing their host specificity (Burazerović et al. 2018; Pejić et al. 2021). So far, 21 ectoparasite species across 18 bat species are documented (Burazerović et al. 2018), emphasizing host-parasite associations without delving into factors affecting ectoparasite abundance and seasonal dynamics. Similarly, high host specificity of bat flies and wing mites on *M. schreibersii* and *Rhinolophus ferrumequinum* is documented for this region, but no ecological or physiological drivers influencing infestation patterns were investigated (Pejić et al. 2021).

This study aims to investigate how seasonal changes and host-related factors—such as reproductive status, sex, age, and body condition—influence the abundance and reproductive dynamics of ectoparasites, specifically ticks, mites, and bat flies, on 3 bat species in the Central Balkans. We hypothesize that ectoparasite abundance varies seasonally, with higher infestations occurring during periods of increased host aggregation and reproductive activity, as well as that host characteristics such as sex, age, reproductive status, and body condition significantly influence total and abundance of life stages indicating reproduction in bat ectoparasites. Ectoparasite abundance (total and specific reproductive stages) serves as the response variable, while seasonality (spring, summer and autumn season) and host traits (sex, age, reproductive status, and body condition) are the predictor variables.

## Materials and methods

### Data collection

#### Sampling sites

The field work was conducted in 2013–2015 during the period of activity of bats (April—October), study being suspended in late May through June during the period of advanced pregnancy, delivery, and first phase of nursing the young bats (Supplementary Tables S1.1A–C in Supplementary Material 1). A total of 29 localities in Serbia, Montenegro, and Bosnia and Herzegovina were investigated during this study (Fig. 1). For *Rhinolophus* species, sampling was conducted at more than 20 sites across the Central Balkans to include as many independent colonies as possible. This broad spatial coverage aimed to capture host-specific patterns of ectoparasite infestation, rather than to test for locality-specific effects. In contrast, sampling of *M. schreibersii* was carried out at 3 maternity colonies and structured as a year-long study with seasonal sampling points. This design enabled us to track within-year changes in ectoparasite abundance and community composition, thereby revealing host-specific seasonal dynamics driven by ecological and physiological factors.

**Figure 1:**
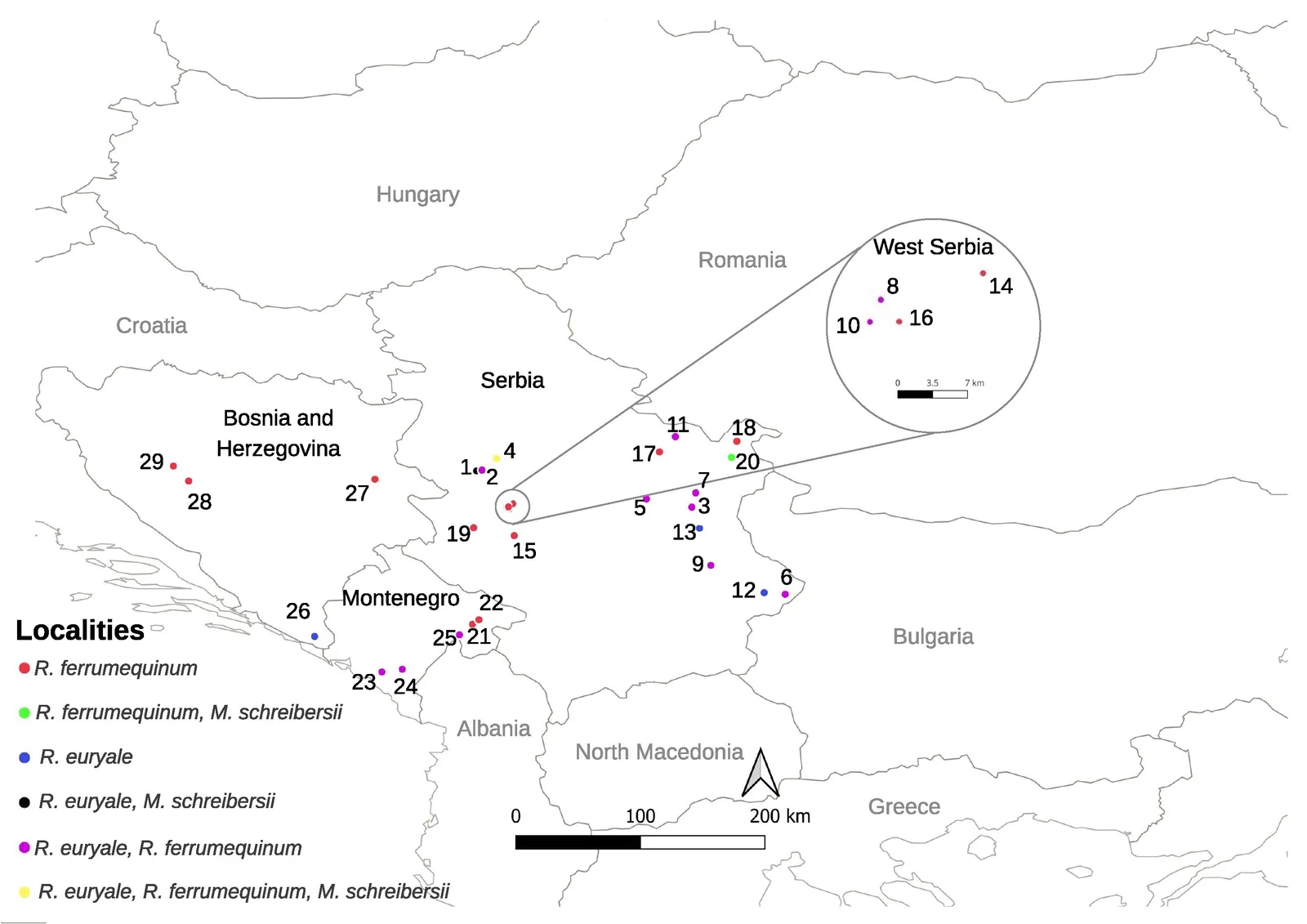
Map with localities where bats and ectoparasites were sampled. Different colors mark localities with caves in which different species of bats (*Rhinolophus ferrumequinum, Rhinolophus euryale, Miniopterus schreibersii*) were captured. (1) Degurićka cave, (2) Petnička cave, (3) Bogovinska cave, (4) Šalitrena cave, (5) Ravanička cave, (6) Petrlaška cave, (7) Zlotska river, (8) Šanički bezdan pit, (9) Ogorelička cave, (10) Kađenica cave, (11) Dubočka cave, (12) Velika cave, (13) Sesalačka cave, (14) Bunker in Ovčar banja, (15) Hadži-Prodanova cave, (16) Old tunnel in Ovčar banja, (17), Ravništarka cave, (18) Sokolovica cave, (19) Stopića cave, (20) Dudićeva cave, (21) Navotinjska cave, (22) Ðato cave, (23) Začirska cave, (24) Šutonjića pit, (25) Osoja cave, (26) Strač fortress, (27) Han-Pjesak, (28) Vaganska cave, (29) Sokolačka cave. Map created in QGIS (v3.36.0, QGIS Development Team 2024), using the Natural Earth dataset (Natural Earth 2020).

#### Capture of bats and collection of ectoparasites

At all localities, bats were caught using a harp trap or a mist net placed at the entrance of the roost, following the best practices guidelines for catching and handling bats (Sedgeley et al. 2013; Collins 2024). Bat capturing started at sunset and was continuous until bat movement was observable. Captured bats were placed separately in clean cotton bags to avoid cross-contamination. Each bat was identified to the species level (Dietz et al. 2009). Age classification (adult or juvenile) and determination of the reproductive stage in females (pregnant, lactating, or nonreproductive) were conducted using standard protocols (Brunet-Rossinni and Wilkinson 2009; Sedgeley et al. 2013). Each bat was measured for body mass and forearm length (Sedgeley et al. 2013). The entire body (wing and tail membranes, fur, face, ears) of each bat host was carefully visually inspected for the presence of ectoparasites: ticks (Acari: Ixodidae), mites (Acarina: Mesostigmata: Macronyssidae, Spinturnicidae), and bat flies (Insecta: Diptera: Nycteribiidae). All ectoparasites found were removed with tweezers and placed in tubes with 70% ethanol and labeled appropriately (locality, date, bat specimen code). All bats were released at the same place where they were caught, immediately after checking for the presence of ectoparasites. Before release, bats were marked with a fur clip on the dorsal side, to avoid pseudo-replication if subsequently recaptured in the same night (Sedgeley et al. 2013).

#### Identification of ectoparasites

All ectoparasites were identified based on morphology to the species level. Ticks and mites were additionally classified by developmental stage and sex. Ticks included larva, nymph and adult stages; mites included nymph (proto- and deutonymph) and adult stages; both groups were divided by sex (female and male). Bat flies were classified by sex only (female and male). Different sexes (in mites, ticks, and bat flies) and developmental stages (mites and ticks) were identified based on morphological differences visible in light microscopy using morphological keys for mites (Stanyukovich 1997; Orlova et al. 2015), nycteribiid bat flies (Karaman 1936; Theodor and Moscona 1954; Theodor 1967), and ticks (Pomerantzev 1950; Arthur 1956; Hornok et al. 2015, 2016). Gravid females in mites can be clearly identified using microscopic slides previously prepared using standard protocols (Stanyukovich 1997), where intrauterine larvae can be observed within the female mites (Orlova et al. 2018). Gravid female bat flies are significantly larger than males, accumulating excess fat reserves, and also exhibit significant abdominal enlargement due to the developing larva, which can be confirmed through dissection (Hutson 1984; Witsenburg 2014; Haelewaters et al. 2017). All identifications were made using a stereo microscope (Zeiss SteREO Discovery.V8) and compound light microscope (Nikon Eclipse E100-LED microscope).

### Data analysis

We used only data where the number of individuals per parasite species sampled was higher than 100 (*Phthiridium biarticulatum* (*N* = 109) in *R. euryale*; *I. simplex* (*N* = 603)*, N. schmidlii* (*N* = 2641), and *S. psi* (*N* = 2306) in *M. schreibersii*), and where there were more than 50 hosts. This threshold was set to ensure adequate sample sizes for reliable statistical analysis. All statistical analyzes were performed in R (v4.3.2, R Core Team 2023).

As factors that can influence the ectoparasite abundance per host individual, we analyzed the following factors in the case of *R. euryale* and *R. ferrumequinum*: sex, body condition index (BCI), and season. We did not use age as a factor in the models of *R.euryale* and *R. ferrumequinum*, because there were not enough juvenile hosts in our sample. In the case of *M. schreibersii,* in accordance with sampling design, we additionally analyzed the effects of host age, and in adult female hosts effects of pregnancy and nursing. The effect of pregnancy was analyzed only during the spring season, while the effect of nursing was analyzed only for the summer season in adult female hosts. Nursing the young of the year was identified in female adults by the presence of visibly active nipples (Sedgeley et al. 2013). Life stages indicating reproduction of bat ectoparasites in this study is represented in (1) bat flies (*N. schmidlii*)—as the number of pregnant females, (2) mites (*S. psi*)—as the number of pregnant female and the number of pre-adult stages (protonymphs and deutonymphs), and (3) ticks (*I. simplex*)—as the number of pre-adult stages (larvae and nymphs).

In all cases, we used GLMs (generalized linear models) with negative binomial distribution (Zuur et al. 2009) (Supplementary Table S1.2 in Supplementary Material 1), from the MASS package (Venables and Ripley 2002), because the data were not normally distributed. The response variable used in the study was ectoparasite abundance per host, measured as: (1) the abundance of ectoparasites, (2) the abundance for specific ectoparasite species, and in the case of *M. schreibersii,* (3) the reproduction activity in different ectoparasite species. Predictor variables included physiological host factors such as sex, age, BCI, and reproductive status (pregnancy and nursing), as well as environmental conditions represented by season. We fitted 6 candidate models for each parasite species, incorporating these predictors either additively or with interactions (Supplementary Tables S1.3 A, B in Supplementary Material 1). Model selection was primarily based on Akaike Information Criterion (AIC) (Zuur et al. 2009) values, with models exhibiting the lowest AIC considered the best fit. To further refine the model choice, Likelihood Ratio Tests (LRT), using the *lmtest* package (Zeileis and Hothorn 2002), were performed among the models with the lowest AIC values to determine which model best suited our data. If the LRT results indicated that there is statistically significant difference, then the model with interaction was applied; otherwise, a model without interaction was applied due to the simplicity of the model. In the case of GLM with interaction, we used a Tukey post hoc from *emmeans* package (Lenth 2024) to more precisely interpret the relationships between categorical variables. For data visualization, we used packages *multcomp* (Hothorn et al. 2008), *tidyverse* (Wickham et al. 2019), and *ggplot2* (Wickham 2016).

In order to better understand the body condition of bats as a factor influencing parameters related to ectoparasitism in bats, we tested which effects influence host BCI using a GLM (Supplementary Tables S1.4, 5 in Supplementary Material 1). BCI was calculated as the ratio of body mass to forearm length (Pearce et al. 2008).

## Results

A total of 15 ectoparasite taxa were identified across 3 bat species, comprising 13 species determined to the species level and 2 identified at the genus level. These included 2 tick species (Ixodidae), 7 bat fly species (Nycteribiidae and Streblidae), and 6 mite taxa (Spinturnicidae and Macronyssidae). In total, 6,435 individual ectoparasites were recorded. The highest prevalence of ectoparasites was recorded in *M. schreibersii* (94.6%), which also exhibited the highest total abundance with 6,056 ectoparasites (see Supplementary Tables S2.1, 2 in Supplementary Material 2). Out of a total of 603 *I. simplex* individuals collected, 66 were adult females, 342 were nymphs, and 195 were larvae; from 2,641 collected Nycteribiidae individuals (*N. schmidlii*), 1,268 were males and 1,373 females (of which 370 were gravid); and for *S. psi*, 867 were males, 996 females (of which 171 were gravid), and 443 nymphs (sample size, prevalence, and total number of ectoparasites in the 3 analyzed species: *M. schreibersii*, *R. euryale*, and *R. ferrumequinum*; Supplementary Table S.2.3, Supplementary Material 2).

### Factors affecting the total abundance of ectoparasites on host species

GLM analyzes revealed several significant factors affecting the total abundance of ectoparasites primarily season, sex, age, BCI, and reproductive status of host species (Supplementary Table S3.1–3 in Supplementary Material 3).

The season proved to be a statistically significant factor for the total abundance of ectoparasites in *R. euryale* and *M. schreibersii* (Table 1). In *R. euryale*, the total abundance of ectoparasites was highest during summer and autumn (*Z* = 2.394, *P* =0.016), and exclusively during summer (*Z* = 3.318, *P* < 0.001) when only *P. biarticulatum* was analyzed. In *M. schreibersii*, the highest total infestation was recorded in spring (*Z* = 13.030, *P* < 0.001) and summer (*Z* = 12.408, *P* < 0.001), compared with autumn, which is also related to the abundance of *I. simplex* (spring: *Z* = 8.312, *P* < 0.001; summer: *Z* = 7.968, *P* < 0.001). The highest abundance of *N. schmidlii* was registered in summer (*Z* = 7.040, *P* < 0.001), while *S. psi* was most abundant in spring (*Z* = 15.569, *P* < 0.001), compared with other seasons.

**Table 1:** Summary of statistically significant predictors of total ectoparasite (ticks, flies, and mites) abundance in *Rhinolophus ferrumequinum*, *Rhinolophus euryale*, and *Miniopterus schreibersii* based on GLM analyzes.

Predictors (Effect)	*Rhinolophus ferrumequinum*	*Rhinolophus euryale*		*Miniopterus schreibersii*			
	Total ectoparasites	Total ectoparasites	Total *Phthiridium biarticulatum*	Total ectoparasites	Total *Ixodes simplex*	Total *Nycteribia schmidli*i	Total *Spinturnix psi*
**Season**	—	Summer = Autumn > Spring	Summer > Spring = Autumn	Spring = Summer > Autumn	Spring = Summer > Autumn	Summer > Autumn > Spring	Spring > Summer > Autumn
**Sex**	Female > Male	—	—	—	Female > Male	—	—
**Age**	—	—	—	—	—	Adult > Juvenile	Juvenile > Adult
**BCI**	—	− correlation	− correlation	+ correlation	—	+ correlation	—
**Pregnancy**	—	—	—	Pregnant > Nonpregnant	Pregnant > Nonpregnant	Pregnant > Nonpregnant	—
**Nursing**	—	—	—	—	Nursing > Non-nursing	—	—

(−), negative correlation; (+), positive correlation; (>), direction of effect; (=), no significant difference between compared levels; BCI, body condition index.

Sex of the host proved to be the only significant factor affecting the total abundance of ectoparasites in *R. ferrumequinum* (*Z* = −3.717, *P* < 0.001). Sex also proved to be an important predictor of *I. simplex* abundance (*Z* = −3.166, *P* = 0.001). Host age had a significant effect on the abundance of *N. schmidlii* and *S. psi* in *M. schreibersii* where adults had more *N. schmidlii* than juveniles (*Z* = −3.575, *P* < 0.001), and juveniles had more *S. psi* than adults (*Z* = 7.821, *P* < 0.001). The effect of BCI was different depending on the host species. In *R. euryale*, BCI had a negative effect on the total abundance of ectoparasites (*Z* = −2.012, *P* = 0.044) and the abundance of *P. biarticulatum* (*Z* = −2.936, *P* < 0.001), while in *M. schreibersii,* a positive effect of BCI was recorded on the total abundance of ectoparasites (*Z* = 2.485, *P* < 0.001) and the abundance of *N. schmidlii* (*Z* = 4.063, *P* < 0.001).

Pregnancy in *M. schreibersii* had a significant positive effect on the total abundance of ectoparasites (*Z* = 2.724, *P* = 0.006). When analyzing the total abundance of individuals for each ectoparasite species, we found that pregnancy had a significant positive effect on the total abundance of *N. schmidlii* (*Z* = 4.041, *P* < 0.001) and the total abundance of *I. simplex* (*Z* = 2.584, *P* = 0.009). Nursing in *M. schreibersii* showed statistically significant positive effects on the total abundance of *I. simplex* (*Z* = 3.978, *P* < 0.001). There were no significant effects of pregnancy (*Z* = 0.404, *P* > 0.05) or nursing (*Z* = 0.001, *P* > 0.05) on the total abundance of *S. psi* in *M. schreibersii.*

In *M. schreibersii*, the total abundance of ectoparasites was influenced by significant interactions between predictors (Supplementary Table S3.4A–D, Supplementary Material 3). Seasonal dynamics and interaction effects on the total abundance of ectoparasites were examined in relation to host sex, age, and season, with patterns also assessed for individual ectoparasite species. Overall, ectoparasite abundance was highest in spring adult females and in summer among adult females and juveniles, with males also showing high infestation in summer juveniles (Fig. 2A). At the species level, *I. simplex* was most abundant in juveniles during summer, *N. schmidlii* in females during summer, while *S. psi* peaked in females during spring (Fig. 2B–D).

**Figure 2:**
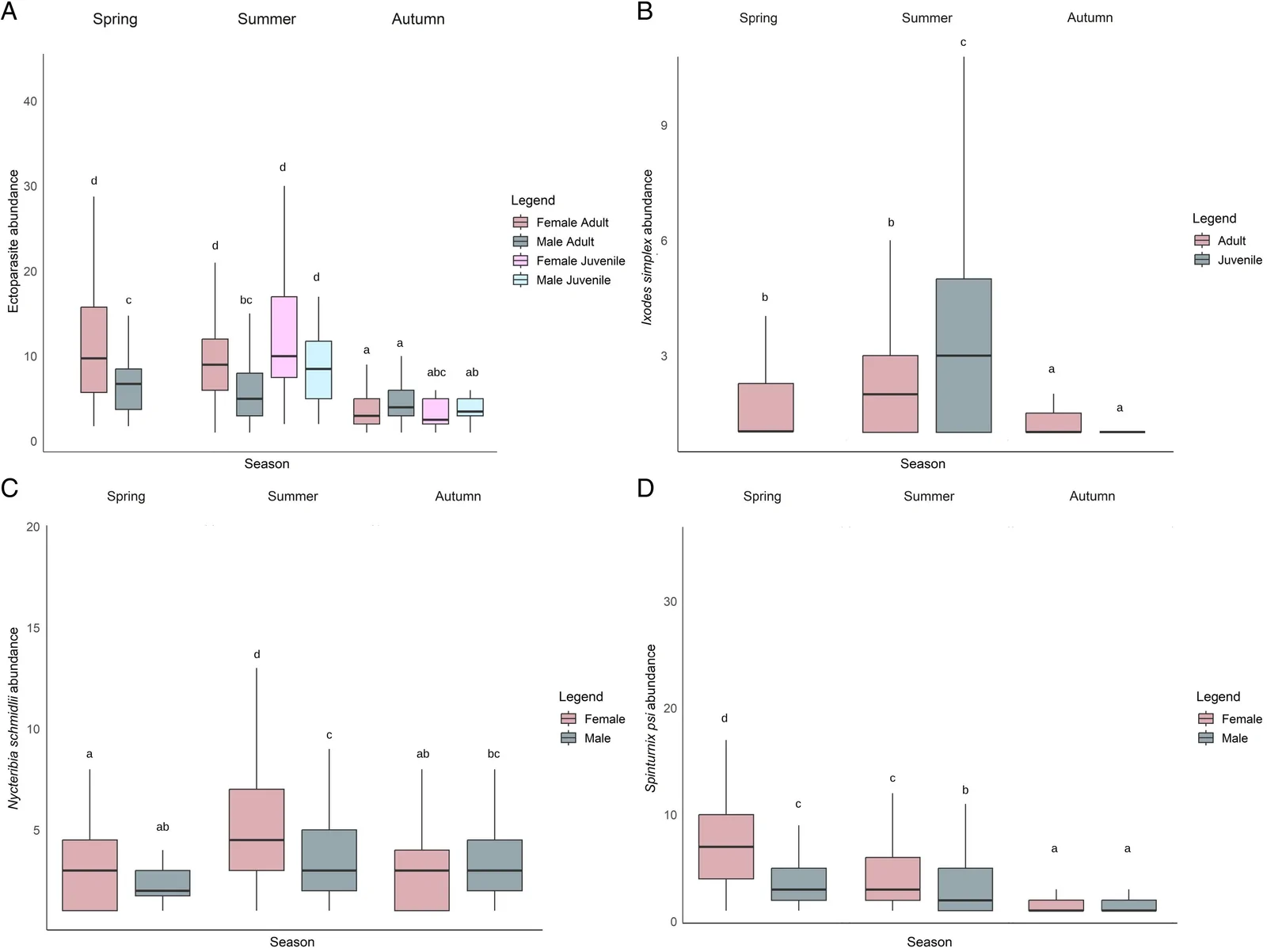
Graphical representation of seasonal dynamics of ectoparasites (ticks, flies, mites) parasitizing *Miniopterus schreibersii* hosts (A) Total abundance of all ectoparasites in hosts of different sexes, ages, and seasons; (B) *Ixodes simplex* total abundance in hosts of different ages and seasons; (C) *Nycteribia schmidlii* total abundance in hosts of different sexes and seasons; (D) *Spinturnix psi* total abundance in hosts of different sexes and seasons. Groups marked with different letters (a–d) are significantly different according to the Tukey post hoc test (*P* < 0.05).

### Factors affecting life stages of *Miniopterus schreibersii* ectoparasites indicative of reproduction and their seasonal dynamics

The life stages of *M. schreibersii* ectoparasites, indicative of reproductive activity, were significantly affected by season, host sex, age, BCI, and reproductive status, according to GLM analyzes (Supplementary Tables S4.1–3, Supplementary Material S4). The season affected the number of developmental stages of all ectoparasites in *M. schreibersii* (Table 2), which indicates a pronounced seasonal dynamic in their reproductive cycle with highest abundance for all ectoparasites in the spring and summer seasons, compared with autumn.

**Table 2:** Summary of statistically significant predictors of life stages indicating reproduction of bat ectoparasites (ticks, flies, mites) in *Miniopterus schreibersii* based on GLM analyzes.

Predictors (Effect)	*Ixodes simplex* female	*Ixodes simplex* nymphs	*Ixodes simplex* larvae	*Nycteribia schmidlii* pregnant female	*Spinturnix psi* pregnant female	*Spinturnix psi* pre-adults
**Season**	Summer > Spring = Autumn	Summer = Spring > Autumn	Summer = Spring > Autumn	Spring > Summer > Autumn	Spring = Summer > Autumn	Spring > Summer > Autumn
**Sex**	—	—	—	Male > Female	Female > Male	—
**Age**	Juvenile > Adult	—	—	—	Juvenile > Adult	Juvenile > Adult
**BCI**	—	+ correlation	—	—	+ correlation	—
**Pregnancy**	—	Pregnant > Nonpregnant	—	—	—	—
**Nursing**	—	Nursing > Non-nursing	Nursing > Non-nursing	Non-Nursing > Nursing	—	—

(+), positive correlation; (>), direction of effect; (=), no significant difference between compared levels; BCI, body condition index.

The sex of the host proved to be a significant factor affecting the abundance of pregnant individuals of *N. schmidlii* (*Z* = 3.786, *P* < 0.001) and *S. psi* (*Z* = −3.093, *P* = 0.001). Host age category also had a significant effect: juveniles showed a higher abundance of *I. simplex* females (*Z* = 6.337, *P* < 0.001), *S. psi* pre-adults (*Z* = 7.351, *P* < 0.001), and pregnant females (*Z* = 3.915, *P* < 0.001). BCI had a positive effect on the abundance of *I. simplex* nymphs (*Z* = 2.940, *P* = 0.003) and pregnant *S. psi* females (*Z* = 2.654, *P* = 0.007). The host reproductive status (pregnancy and/or nursing) significantly increased the number of *I. simplex* nymphs (pregnancy: *Z* = 2.946, *P* < 0.001; nursing: *Z* = 3.897, *P* < 0.001) and larvae (nursing: *Z* = 2.651, *P* = 0.008), and conversely decreased the number of pregnant *N. schmidlii* females (nursing: *Z* = −3.289, *P* = 0.001).

Significant interactions between sex and season of the host were found for the abundance of *I. simplex* larvae, pregnant individuals of *N. schmidlii*, and pre-adult stage of *S. psi*, which indicates that the seasonal dynamics of these developmental stages differed depending on the host sex. The abundance of *I. simplex* larvae was significantly higher in female hosts during summer and spring compared with other groups. Male hosts in all seasons and female hosts in autumn showed similarly low numbers, without statistically significant differences between them (Supplementary Table S4.4A, Supplementary Material S4). The highest number of pregnant females of *N. schmidlii* is in the spring and summer, in particular in female hosts during the spring season (Supplementary Table S4.4B in Supplementary Material S4). In spring, female hosts have more of *S. psi* pre-adult forms, in comparison to male and female hosts in summer, while in autumn is generally less than in spring and summer (Supplementary Table S4.4C in Supplementary Material S4).

## Discussion

### Season as a factor affecting abundance of bat ectoparasites in the Central Balkans

Throughout the year, bat colony structure and aggregation patterns in Europe undergo marked seasonal changes, primarily driven by reproductive cycles and environmental conditions (Dietz et al. 2009). During spring and summer, many bat species, such as *M. schreibersii*, *R. euryale*, and *R. ferrumequinum*, form large maternity colonies composed predominantly of reproductive females, which cluster closely together to rear their young (Dietz et al. 2009). In contrast, males and nonreproductive individuals are often found in smaller groups or roosting solitarily (Dietz et al. 2009). As autumn approaches and reproductive activity ceases, maternity colonies disband, and individuals gradually shift to hibernation roosts. Hibernation occurs exclusively during the winter months in cold, humid underground sites (Dietz et al. 2009). During this period, ectoparasite activity typically decreases significantly, as many ectoparasite species reduce their numbers and reproductive activity (Lourenço and Palmeirim 2008). These seasonal shifts in colony structure and host physiology directly influence the transmission dynamics and prevalence of ectoparasites (Lourenço and Palmeirim 2008; van Schaik and Kerth 2017).

Our results strongly support the role of seasonality as a key factor influencing bat ectoparasite abundance in the Central Balkans, likely driven by the formation of maternity colonies and increased host density during the reproductive season (Lourenço and Palmeirim 2008; van Schaik and Kerth 2017). Greater ectoparasite loads are consistently associated with females, and not with males, throughout the reproductive season (see Tables 1 and 2). We found the highest numbers of ectoparasites in both *R. euryale* and *M. schreibersii* during the formation of maternity colonies, which consist of adult females and their offspring. This period represents a critical window for parasite transmission due to increased host density, stable microclimatic conditions, and prolonged social contact, all of which facilitate parasite establishment and reproduction (Lourenço and Palmeirim 2007; Reckardt and Kerth 2007). Results of our study suggest that seasonality, mediated through sex-specific reproductive behavior and social structure, is a major driver of ectoparasite dynamics in this host-parasite system (van Schaik and Kerth 2017).

In *R. euryale*, ectoparasite numbers were highest in summer and autumn (Table 1), while spring had noticeably lower infestation levels. This pattern suggests that the warmer and more stable environmental conditions during summer and autumn may promote parasite survival and reproduction, in contrast to the cooler and possibly less favorable conditions (Lourenço and Palmeirim 2007). Specifically, *P. biarticulatum* reaches its highest numbers in summer (Table 1), likely benefiting from the warm and humid microclimate within maternity roosts, which supports its reproductive cycle and development (Zahn and Rupp 2004). More detailed investigations of seasonal dynamics of the *R. euryale* ectoparasites should be the topic of future research.

At the level of individual ectoparasite species parasitizing *M. schreibersii*, we can generally say that the highest abundance, for each of the 3 analyzed species of ectoparasites, is found during the spring and summer seasons, when the density of the host colonies are highest in maternity colonies (personal observation from the field). These colonies are made of female bats (in spring) or female and juvenile bats (in summer). Therefore, it is not surprising that the highest numbers of ectoparasites are found either in female hosts during the spring (*S. psi,*  Fig. 2D) and summer season (*N. schmidlii,*  Fig. 2C), or in juveniles during the summer season (*I. simplex,*  Fig. 2B). For all 3 analyzed parasite species, the lowest number of parasites are found during the autumn (Fig. 2B–D), the result which is also in accordance with the total number of ectoparasites on *M. schreibersii*. This may be caused by the fact that in autumn colonies of this host species disperse and the transmission of ectoparasites between host individuals is at much lower rate compared with spring and summer seasons (Lourenço and Palmeirim 2007).

In the neighboring countries of Romania and Bulgaria, *I. simplex* is more common in spring and summer, with decreasing numbers toward autumn (Sándor et al. 2019). In our study, *I. simplex* exhibits the same seasonal dynamics, with the addition that it is more numerous on female hosts compared with male hosts, and that juveniles in summer host the highest numbers of this parasite. Our findings align with the limited research on seasonal and physiological drivers of bat ectoparasite dynamics, particularly in European bat species. In European bats, high numbers of Spinturnicidae and Nycteribiidae have been observed during the summer nursery period (Zahn and Rupp 2004). Similar seasonal trends are reported globally: in China, ectoparasite loads in vesper bats decline from spring to winter (Zhang et al. 2010), while in Iran, *Pipistrellus kuhlii* exhibits seasonal fluctuations in parasite burden, decreasing from spring to winter (Sharifi et al. 2018). Our findings underscore the seasonal dynamics of bat ectoparasite infestations, with peak burdens aligning with the summer nursery period. This pattern suggests that host reproductive cycles and environmental factors are key drivers of ectoparasite abundance across species and geographic regions.

In *M. schreibersii* in our study, the reproduction of ectoparasite species is synchronized with the reproduction cycle of their host and happens when the host colonies of this species are densest and made up of females (spring season) or females and juveniles (summer season). The reproduction of *N. schmidlii* happens during the spring, *S. psi* in spring and summer seasons, while pre-adult development stages of *I. simplex* are most abundant in spring and summer seasons as well. The pregnant parasites and pre-adult forms are generally more abundant on female hosts (*N. schmidlii,*  Supplementary Table S4.4B) or female and juvenile hosts (*S. psi,*  Supplementary Table S4.4C). Some of our findings related to reproduction and development activity of the 3 ectoparasite species are in accordance with the famous study of Lourenço and Palmeirim (2008) that focuses on life stages indicating reproduction of ectoparasites and their abundance more than different seasons in Portugal. For the 2 out of 3 analyzed species of ectoparasites in our research, the results are almost identical in both geographical regions: *I. simplex* shows the highest numbers of pre-adult stages in spring and summer seasons; *N. schmidlii* shows the highest numbers of pregnant individuals in female hosts in spring; and *S. psi* shows the highest numbers of pregnant individuals in juvenile hosts. However, our results indicate that the reproduction period of *S. psi* starts earlier in the region of the Central Balkans, with the highest numbers of pregnant *S. psi* females are found in spring, while in Portugal the highest number is in the summer during the nursing season (Lourenço and Palmeirim 2008). The earlier start of reproduction of bat mites in Serbia compared with Portugal is likely due to a combination of climatic and ecological factors, to be investigated in more detail in the future. The Central Balkans (including Serbia where *M. schreibersii* roosts were sampled) and Portugal have different climatic conditions, with Serbia experiencing a continental climate and Portugal having a Mediterranean climate (Beck et al. 2018). These climate differences can influence the timing of reproduction in ectoparasites. Serbia's colder winters and shorter but warmer summers might trigger mites to start reproducing earlier in spring to maximize their reproductive success before colder conditions set in, as the reproduction of this parasite completely stops during the winter season (Lourenço and Palmeirim 2008). In contrast, the milder temperatures in Portugal may result in a slightly delayed start to reproduction, as the environmental cues for reproduction (e.g., temperature or humidity) are different.

The lack of differences in the timing of development and reproduction between bat ticks and bat flies in Serbia and Portugal, combined with earlier reproduction of bat mites in Serbia, suggests that different ectoparasite species may be regulated by different ecological, environmental, and biological factors (Lourenço and Palmeirim 2008; da Silva et al. 2023). Bat ticks and flies, possibly influenced more by host behavior and bat colony dynamics (Reckardt and Kerth 2007), may remain synchronized in both regions despite climatic differences (Beck et al. 2018). In contrast, bat mites, which are more sensitive to temperature and microclimatic conditions, may show earlier reproduction in Serbia due to regional differences in climate, host behavior, and environmental cues. Understanding these subtle differences in host-parasite interactions can provide valuable insights into the ecological dynamics of ectoparasites under changing environmental conditions.

### Host traits (sex, age, and BCI) as factors affecting abundance of bat ectoparasites in the Central Balkans

Our results suggest that host sex influences ectoparasite load in *R. ferrumequinum*, with females harboring significantly more parasites than males (Table 1). A similar sex-biased parasitism was observed in *M. schreibersii*, specifically for *I. simplex*. This pattern aligns with findings from several studies indicating higher ectoparasite burdens in female bats, particularly during maternity periods when individuals form large, stable colonies and experience increased contact rates, humidity, and roost fidelity, all of which may facilitate parasite transmission (Zahn and Rupp 2004; Christe et al. 2007).

Age significantly influenced the abundance of *N. schmidlii* and *S. psi* in *M. schreibersii*, but in opposite directions (Table 1). Adults harbored more *N. schmidlii* than juveniles, while *S. psi* was more abundant in juveniles. These contrasting patterns likely reflect differences in the biology and ecology of the 2 ectoparasite taxa. Wing mites such as *S. psi* might be transmitted early in life, particularly in dense maternity roosts where close contact facilitates spread among juveniles, who also have immature immune defenses and less efficient grooming behavior (Christe et al. 2001; Lourenço and Palmeirim 2007). In contrast, *N. schmidlii*, a wing-dwelling nycteribiid fly, may show a preference for adult hosts due to their more stable roosting behavior, larger body size, and greater mobility, which could aid in parasite dispersal and survival (Zahn and Rupp 2004). Since juveniles tend to have lower survival rates during winter compared with adults (Postawa and Nagy 2016), adult hosts may represent a more reliable option for parasite persistence. These results highlight how host age interacts with parasite life history traits to shape infestation dynamics.

The BCI significantly influences ectoparasite abundance in *R. euryale* and *M. schreibersii* in contrasting directions; in *R. euryale*, ectoparasite load increases as BCI decreases, whereas in *M. schreibersii*, ectoparasite numbers rise with increasing BCI. Additionally, it is positively correlated with the total number of *N. schmidlii*. When looking at stages of development that are associated with reproduction in ectoparasites, BCI positively affects development and reproduction of *I. simplex* and *S.psi*, which could reflect the synchronization of life cycles between the host and the parasite, in the case of *M. schreibersii* and its ectoparasites.

This relationship is potentially bidirectional and complex—parasites can reduce host condition, while host condition may affect susceptibility or attractiveness to parasites, resulting in different infestation patterns across species (Sánchez et al. 2018). Studies examining the influence of body condition on ectoparasite load in European bats remain limited and report contrasting results. A negative correlation has been reported between mite load (*S. psi*) and body condition in *M. schreibersii*, though no data are available on the relationship between total ectoparasite load and bat body condition (Lourenço and Palmeirim 2007). In a broader study of European bats (excluding the species investigated in our research), no consistent trend linking decreasing BCI with increased parasite load was found (Zahn and Rupp 2004). This study suggested that the high number of parasites in weakened bats is more likely a symptom of poor condition than a contributing factor. Similarly, in central Anatolia no association between BCI and the intensity of infection by *S. psi*, *N. schmidlii*, and *Penicillidia dufourii* in *M. schreibersii* was found (Postawa and Furman 2014). In contrast, a study on *R. madagascariensis* in Madagascar indicated a weak positive relationship between host BCI and ectoparasite abundance, with parasites correlating positively with reproductive state and female-biased parasitism (Rajemison et al. 2017). In Taiwan region, research on *Miniopterus fuliginosus* demonstrated that reproductive females, particularly lactating individuals, exhibited better body condition and higher ectoparasite infestations, including nycteribiid and streblid bat flies and wing mites of the genus *Spinturnix* (Tai et al. 2022). These findings are in accordance with results of our study concerning the connection between BCI and the number of ectoparasites in one of our studied species, *M. schreibersii*, as a host species. Weak correlations between host body condition and total ectoparasite abundance was found in some recent European studies (Péter et al. 2025); however, when examining parasite groups separately, patterns diverged: wing mite numbers increased significantly with better host condition, while nycteribiid bat flies were less prevalent and less abundant in individuals in good body condition. In contrast, tick infestation appeared unrelated to host condition. However, this study did not examine different stages indicating reproduction in bat ectoparasites, thus cannot be directly compared with results of our study. Future studies using controlled infestation experiments could offer deeper insights into bat–parasite interactions, though they require specialized lab conditions. Highlighting these challenges aims to support and inspire further research on how ectoparasite dynamics relate to bat health, reproductive status, and ectoparasite success across different regions and species. Based on the available literature, the relationship between bat body condition and ectoparasite load is complex and context-dependent; while some studies have reported a negative correlation between host body condition and ectoparasite load, others suggest that the abundance of ectoparasites may be more closely linked to the reproductive state, sex, or species-specific characteristics of the host (Lourenço and Palmeirim 2007; Rajemison et al. 2017; Tai et al. 2022). Further research is needed to unravel the complex dynamics between bat health, reproductive status, and ectoparasite success across different regions and bat species.

Our results show that reproductive status of *M. schreibersii* significantly modulates ectoparasite burden, with pregnancy associated with increased total ectoparasite load, including higher numbers of *I. simplex*—especially nymphs—and *N. schmidlii* (Tables 1 and 2). This aligns with evidence that pregnancy in bats can suppress immune function, increase metabolic stress, and prolong roost fidelity, all contributing to elevated susceptibility to parasitism (Christe et al. 2001; Klein 2004). Pregnant females typically cluster in dense maternity colonies during spring and summer, facilitating horizontal transmission of ectoparasites. During nursing, however, we observed an increase in *I. simplex* (notably nymphs and larvae) but a reduction in gravid *N. schmidlii*, suggesting differential responses among parasite taxa to host reproductive status, possibly due to behavioral changes like increased grooming or shifts in physiological cues (Zahn and Rupp 2004). In addition to reproductive status, host sex, age, and body condition also significantly influenced ectoparasite abundance, highlighting how seasonal dynamics and host traits jointly shape parasite patterns. These findings emphasize the complexity of host-parasite interactions shaped by host biology, social structure, parasite life history strategies, and environmental context, underscoring the need to consider both host and parasite ecology to better understand their interactions in wild bat populations. This study fills a critical gap in our understanding of bat ectoparasite ecology in the Central Balkans, highlighting the importance of regional and species-specific factors in shaping parasite communities and providing a foundation for future research and conservation efforts in this understudied region.

## Supplementary Material

zoaf079_Supplementary_Data

## Data Availability

The datasets generated during and/or analyzed during the current study are available from the corresponding author on reasonable request.
