# Supplementary material for: Seasonal patterns and host traits as drivers of ectoparasite abundance in 3 bat species (Miniopterus schreibersii, Rhinolophus euryale, and Rhinolophus ferrumequinum) in the Central Balkans
Source: Curr Zool. 2025 Dec 22;72(5):692–702. doi: 10.1093/cz/zoaf079 (PMC13690573; doi:10.1093/cz/zoaf079)
Supplement: Supplementary file 1 [file zoaf079_supplementary_data.zip › Supplementary Material 1 R3.docx]

**Supplementary Material 1**

**Table S1.1.** Detailed sampling effort per bat species, roost, and season, including number of samples, nights, and sampling months.

**Table S1.1a:** Number of samples (examined bat individuals), sampling nights, and months per roost, season for *Rhinolophus ferrumequinum.*

| **Locality** | **Season** | **Number of samples** | **Number of nights** | **Sampling months** |
| --- | --- | --- | --- | --- |
| **Bogovinska cave** | Spring | 17 | 1 | April |
|  | Summer | 1 | 1 | May |
| **Bunker** **Bunker in Ovčar banja** | Summer | 1 | 1 | August |
| **Đato** | Autumn | 5 | 1 | September |
| **Dubočka cave** | Summer | 12 | 1 | July |
| **Dudićeva cave** | Summer | 2 | 1 | July |
|  | Autumn | 3 | 1 | September |
| **Hadži Prodanova cave** | Spring | 2 | 1 | May |
| **Han Pjesak** | Autumn | 2 | 1 | September |
| **Šutonjica pit** | Autumn | 7 | 1 | September |
| **Kađenica cave** | Summer | 1 | 1 | July |
| **Navotinjsko cave** | Autumn | 3 | 1 | September |
| **Ogorelička cave** | Spring | 6 | 1 | April |
| **Osoja cave** | Autumn | 2 | 1 | September |
| **Petnička cave** | Spring | 4 | 1 | May |
| **Petrlaška cave** | Autumn | 6 | 1 | September |
| **Ravanička cave** | Spring | 6 | 1 | April |
|  | Summer | 1 | 1 | July |
|  | Autumn | 4 | 1 | September, October |
| **Ravanštarka cave** | Summer | 1 | 1 | July |
| **Šalitrena cave** | Spring | 1 | 1 | April |
|  | Summer | 1 | 1 | August |
|  | Autumn | 5 | 2 | September, October |
| **Šanički bezdan pit** | Summer | 3 | 2 | July, August |
| **Sokolačka cave** | Autumn | 2 | 1 | September |
| **Sokolovica** | Summer | 1 | 1 | August |
| **Old tunel in Ovčar banja** | Autumn | 2 | 1 | October |
| **Stopića cave** | Summer | 1 | 1 | August |
| **Vaganska cave** | Autumn | 8 | 1 | September |
| **Vilina cave** | Autumn | 1 | 1 | September |
| **Začirska cave** | Autumn | 4 | 1 | September |
| **Zlotska river** | Summer | 4 | 1 | May |

**Table S1.1b:** Number of samples (examined bat individuals), sampling nights, and months per roost, season for *Rhinolophus euryale.*

| **Locality** | **Season** | **Number of samples** | **Number of nights** | **Sampling months** |
| --- | --- | --- | --- | --- |
| **Bogovinska cave** | Spring | 1 | 1 | April |
|  | Summer | 1 | 1 | May |
| **Degurićka cave** | Spring | 1 | 1 | May |
|  | Autumn | 3 | 1 | September |
| **Dubočka cave** | Summer | 5 | 1 | July |
| **Šutonjića pit (Grbavci village)** | Autumn | 2 | 1 | September |
| **Kađenica cave** | Summer | 1 | 1 | July |
| **Ogorelička cave** | Spring | 11 | 1 | April |
| **Osoja cave** | Autumn | 2 | 1 | September |
| **Petnička cave** | Spring | 11 | 1 | May |
|  | Summer | 1 | 1 | August |
| **Petrlaška cave** | Spring | 1 | 1 | May |
|  | Autumn | 5 | 1 | September |
| **Ravanička cave** | Spring | 2 | 1 | April |
|  | Summer | 10 | 2 | July, August |
| **Šalitrena cave** | Spring | 3 | 1 | April |
|  | Summer | 1 | 1 | August |
|  | Autumn | 6 | 2 | September, October |
| **Šanički bezdan pit** | Summer | 28 | 2 | July, August |
|  | Autumn | 3 | 1 | October |
| **Sesalačka cave** | Summer | 1 | 1 | July |
| **Strač fortress** | Autumn | 7 | 1 | September |
| **Velika cave** | Autumn | 24 | 1 | September |
| **Začirska cave** | Autumn | 23 | 1 | September |
| **Zlotska river** | Summer | 3 | 1 | May |

**Table S1.1c:** Number of samples (examined bat individuals), sampling nights, and months per roost, season for *Miniopterus schreibersii*.

| **Locality (Roost)** | **Season** | **Number of samples** | **Number of nights** | **Sampling months** |
| --- | --- | --- | --- | --- |
| **Dudićeva cave** | Spring | 53 | 1 | May |
|  | Summer | 67 | 1 | July |
|  | Autumn | 72 | 1 | September |
| **Ravanička cave** | Spring | 95 | 2 | April, May |
|  | Summer | 128 | 2 | July, August |
|  | Autumn | 127 | 3 | September, October |
| **Šalitrena cave** | Spring | 93 | 3 | April, May |
|  | Summer | 140 | 2 | July, August |
|  | Autumn | 114 | 2 | September, October |

Tables S1.2 and S1.3 provide a detailed overview of the main statistical analyses conducted in this study. The response variable was ectoparasite abundance per host, assessed as: (1) the total abundance of ectoparasites, (2) the total abundance of specific ectoparasite species, and, in the case of *M. schreibersii*, (3) the abundance of life stages indicative of reproductive activity in different ectoparasite taxa. Predictor variables included physiological host factors—sex, age, body condition index (BCI), and reproductive status (pregnancy and nursing)—as well as environmental conditions represented by season.

**Table S1.2** Summary of parasite distribution parameters including Shapiro-Wilk normality test *P* - values (S-W test), mean abundance ± standard deviation (SD), variance, and distributional graphics (density plots) for each parasite taxon.

| **Parasites (tick, flies, mites)** | **S-W test *P* - value** | **Mean ± SD** | **Variance** | | **Distribution plot** |
| --- | --- | --- | --- | --- | --- |
| ***Rhinolophus ferrumequinum*** | | | | | |
| Parasites Total | < 0.001 | 1.47 ± 0.24 | | 6.95 |  |
| ***Rhinolophus euryale*** | | | | | |
| Parasites Total | < 0.001 | 1.32 ± 0.21 | | 6.158 |  |
| *Phthiridium biarticulatum* Total | < 0.001 | 0.71 ± 0.1 | | 1.410 |  |
| ***Miniopterus schreibersii*** | | | | | |
| Parasites Total | < 0.001 | 6.81 ± 0.2 | | 35.814 |  |
| *Ixodes simplex* Total | < 0.001 | 0.68 ± 0.05 | | 2.18 |  |
| *Ixodes simplex* females | < 0.001 | 0.07 ± 0.01 | | 0.19 |  |
| *Ixodes simplex* nymphs | < 0.001 | 0.38 ± 0.03 | | 0.912 |  |
| *Ixodes simplex* larvae | < 0.001 | 0.22 ± 0.02 | | 0.522 |  |
| *Nycteribia schmidlii* Total | < 0.001 | 2.97 ± 0.1 | | 8.291936 |  |
| *Nycteribia schmidlii* pregnant females | < 0.001 | 0.65 ± 0.04 | | 1.097 |  |
| *Spinturnix psi* Total | < 0.001 | 4.59 ± 0.22 | | 18.873 |  |
| *Spinturnix psi* pregnant females | < 0.001 | 0.488 ± 0.05 | | 0.7206 |  |
| *Spinturnix psi* pre-adults | < 0.001 | 0.5 ± 0.04 | | 1.70 |  |

**Table S1.3** Summary of tested GLM models with predictors and model selection criteria (AIC, ΔAIC). ΔAIC (Delta AIC) is the difference between each model’s AIC and the lowest AIC among the tested models. Models with ΔAIC ≤ 2 are considered equally supported. **Bolded models***** were chosen for further analysis based on AIC comparison and Likelihood Ratio Tests (LRT) against models with the lowest AIC.

**Table S1.3a**: Summary of tested GLM models with predictors and model selection criteria (AIC, ΔAIC) in *Rhinolophus ferrumequinum* and *R. euryale*.

| Response variable: Parasites Total in *Rhinolophus ferrumequinum* | | | |
| --- | --- | --- | --- |
| Model | Predictors | AIC | ΔAIC |
| **M1***** | **Sex + Season + BCI** | **363.87** | **0** |
| M2 | Sex * Season + BCI | 363.95 | 0.09 |
|  |  |  |  |
| Response variable: Parasites Total in *Rhinolophus euryale* | | | |
| Model | Predictors | AIC | ΔAIC |
| **M1***** | **Sex + Season + BCI** | **457.14** | **0** |
| M2 | Sex * Season + BCI | 458.65 | 1.51 |
| Response variable: *Phthiridium biarticulatum* in *Rhinolophus euryale* | | | |
| Model | Predictors | AIC | ΔAIC |
| **M1***** | **Sex + Season + BCI** | **334.66** | **0** |
| M2 | Sex * Season + BCI | 337.43 | 2.77 |

**Table S1.3b**: Summary of tested GLM models with predictors and model selection criteria (AIC, ΔAIC) in *Miniopterus schreiberii*.

| Response variable: Parasites Total | | | |
| --- | --- | --- | --- |
| Model | Predictors | AIC | ΔAIC |
| M1 | Sex + Age + Season + BCI | 4931.35 | 39.71 |
| **M2***** | **Sex * Age * Season + BCI** | **4891.64** | **0** |
| M3 | Age * Sex * Season + BCI | 4891.64 | 0 |
| M4 | Sex * Season + Age + BCI | 4899.64 | 8 |
| M5 | Age + BCI + Sex * Season | 4899.64 | 8 |
| M6 | Age * Season + Sex + BCI | 4925.3 | 33.66 |
| Response variable: *Ixodes simplex* Total | | | |
| Model | Predictors | AIC | ΔAIC |
| M1 | Sex + Age + Season + BCI | 1789.81 | 2.03 |
| M2 | Sex * Age * Season + BCI | 1791 | 3.23 |
| M3 | Age * Sex * Season + BCI | 1791 | 3.23 |
| M4 | Sex * Season + Age + BCI | 1787.77 | 0 |
| M5 | Age + BCI + Sex * Season | 1787.77 | 0 |
| **M6***** | **Age * Season + Sex + BCI** | **1790.77** | **2.99** |
| Response variable: *Ixodes simplex* female | | | |
| Model | Predictors | AIC | ΔAIC |
| **M1***** | **Sex + Age + Season + BCI** | **338.55** | **2.56** |
| M2 | Sex * Age * Season + BCI | 343.46 | 7.48 |
| M3 | Age * Sex * Season + BCI | 343.46 | 7.48 |
| M4 | Sex * Season + Age + BCI | 342.5 | 6.52 |
| M5 | Age + BCI + Sex * Season | 342.5 | 6.52 |
| M6 | Age * Season + Sex + BCI | 335.98 | 0 |
| Response variable: *Ixodes simplex* nymphs | | | |
| Model | Predictors | AIC | ΔAIC |
| **M1***** | **Sex + Age + Season + BCI** | **1312.43** | **0.98** |
| M2 | Sex * Age * Season + BCI | 1316.84 | 5.4 |
| M3 | Age * Sex * Season + BCI | 1316.84 | 5.4 |
| M4 | Sex * Season + Age + BCI | 1311.44 | 0 |
| M5 | Age + BCI + Sex * Season | 1311.44 | 0 |
| M6 | Age * Season + Sex + BCI | 1314.28 | 2.83 |
| Response variable: *Ixodes simplex* larvae | | | |
| Model | Predictors | AIC | ΔAIC |
| M1 | Sex + Age + Season + BCI | 916.6 | 2.72 |
| M2 | Sex * Age * Season + BCI | 916.72 | 2.85 |
| M3 | Age * Sex * Season + BCI | 916.72 | 2.85 |
| **M4***** | **Sex * Season + Age + BCI** | **913.87** | **0** |
| M5 | Age + BCI + Sex * Season | 913.87 | 0 |
| M6 | Age * Season + Sex + BCI | 918.4 | 4.52 |
| Response variable: *Nycteribia schmidlii* Total | | | |
| Model | Predictors | AIC | ΔAIC |
| M1 | Sex + Age + Season + BCI | 3836.16 | 9.1 |
| M2 | Sex * Age * Season + BCI | 3830.13 | 3.07 |
| M3 | Age * Sex * Season + BCI | 3830.13 | 3.07 |
| **M4***** | **Sex * Season + Age + BCI** | **3827.06** | **0** |
| M5 | Age + BCI + Sex * Season | 3827.06 | 0 |
| M6 | Age * Season + Sex + BCI | 3838.15 | 11.09 |
| Response variable: *Nycteribia schmidlii* pregnant | | | |
| Model | Predictors | AIC | ΔAIC |
| M1 | Sex + Age + Season + BCI | 1183.39 | 16.71 |
| M2 | Sex * Age * Season + BCI | 1171.04 | 4.36 |
| M3 | Age * Sex * Season + BCI | 1171.04 | 4.36 |
| **M4***** | **Sex * Season + Age + BCI** | **1166.68** | **0** |
| M5 | Age + BCI + Sex * Season | 1166.68 | 0 |
| M6 | Age * Season + Sex + BCI | 1183.21 | 16.53 |
| Response variable: *Spinturnix psi* Total | | | |
| Model | Predictors | AIC | ΔAIC |
| M1 | Sex + Age + Season + BCI | 3247.72 | 16.65 |
| M2 | Sex * Age * Season + BCI | 3231.25 | 0.18 |
| M3 | Age * Sex * Season + BCI | 3231.25 | 0.18 |
| **M4***** | **Sex * Season + Age + BCI** | **3231.07** | **0** |
| M5 | Age + BCI + Sex * Season | 3231.07 | 0 |
| M6 | Age * Season + Sex + BCI | 3249.39 | 18.32 |
| Response variable: *Spinturnix psi* pregnant | | | |
| Model | Predictors | AIC | ΔAIC |
| **M1***** | **Sex + Age + Season + BCI** | **628.79** | **0** |
| M2 | Sex * Age * Season + BCI | 637.65 | 8.85 |
| M3 | Age * Sex * Season + BCI | 637.65 | 8.85 |
| M4 | Sex * Season + Age + BCI | 632.21 | 3.42 |
| M5 | Age + BCI + Sex * Season | 632.21 | 3.42 |
| M6 | Age * Season + Sex + BCI | 630.75 | 1.96 |
| Response variable: *Spinturnix psi* pre-adults | | | |
| Model | Predictors | AIC | ΔAIC |
| M1 | Sex + Age + Season + BCI | 1295.49 | 7.15 |
| M2 | Sex * Age * Season + BCI | 1288.34 | 0 |
| M3 | Age * Sex * Season + BCI | 1288.34 | 0 |
| **M4***** | **Sex * Season + Age + BCI** | **1291.74** | **3.4** |
| M5 | Age + BCI + Sex * Season | 1291.74 | 3.4 |
| M6 | Age * Season + Sex + BCI | 1292.98 | 4.64 |

Statistical details related to the additional analysis of BCI are summarized in Tables S1.4 and S1.5, including descriptive parameters, graphical representations of data distribution, and model outputs with corresponding statistical metrics.

**Table S1.4** Descriptive summary of the body condition index (BCI) per host species. The table includes the *P*-values of the Shapiro–Wilk normality test (S-W test), mean ± standard deviation (SD), variance, and graphical representation of BCI distribution (density plot) for each species.

| **Host bat species** | **S-W test *P* - value** | **Mean ± SD** | **Variance** | **Distribution plot** |
| --- | --- | --- | --- | --- |
| *Rhinolophus ferrumequinum* | < 0.001 | 0.39 ± 0.01 | 0.006 |  |
| *Rhinolophus euryale* | 0.216 | 0.24 ± 0.00 | 0.008 |  |
| *Miniopterus schreibersii* | < 0.001 | 0.28 ± 0.00 | 0.008 |  |

**Table S1.5** Results of GLMs to assess the effects of sex and age on **body condition index (BCI)** in *Rhinolophus euryale, Rhinolophus ferumequinum*, and  *Miniopterus schreibersii*. For each host, a GLM was performed on three samples: the entire sample, the sample of female hosts that are pregnant or lactating, and the sample from which females in reproductive status were excluded. (JUV – juvenile host, M - male host, Nipples Y – female host who has breastfed, Pregnant Y – pregnant female host, E - estimate, SE – standard error, Z- value of Z statistics, *P* - value of the test; statistical significance: *P* < 0.05, n.s. – not statistically significant)*.*

| **Host and sample type** | **Variable** | **Estimate** | **Std. Error** | **Z value** | ***P* -value** |
| --- | --- | --- | --- | --- | --- |
| *R. euryale* entire sample | (Intercept) | -1.416 | 0.014 | -98.655 | **<0.001** |
|  | **GenderM** | -0.045 | 0.019 | 2.999 | **0.022** |
| *R. euryale* nursing/no-nursing females | (Intercept) | -1.434 | 0.016 | -85.792 | **<0.001** |
|  | **NipplesY** | 0.083 | 0.027 | 3.091 | **0.003** |
| *R. euryale* without nursing females | (Intercept) | -1.452 | 0.017 | -81.965 | **<0.001** |
|  | GenderM | -0.009 | 0.022 | -0.414 | n.s. |
| *R. ferrumequinum* entire sample | (Intercept) | -0.915 | 0.026 | -35.05 | **<0.001** |
|  | GenderM | -0.068 | 0.036 | -1.86 | n.s. |
| *R. ferrumequinum* nursing/no-nursing females | (Intercept) | -0.991 | 0.037 | -26.12 | **<0.001** |
|  | **NipplesY** | 0.167 | 0.053 | 3.16 | 0.003 |
| *R. ferrumequinum* without nursing females | (Intercept) | -0.982 | 0.033 | -29.042 | **<0.001** |
|  | GenderM | -0.002 | 0.042 | -0.035 | n.s. |
| *M. schreibersii* entire sample | (Intercept) | -1.232 | 0.004 | -271.842 | **<0.001** |
|  | **GenderM** | -0.039 | 0.006 | -6.023 | **<0.001** |
|  | **AgeJUV** | -0.046 | 0.009 | -4.832 | **<0.001** |
| *M. schreibersii* pregnant/no-pregnant females | (Intercept) | -1.278 | 0.008 | -150.82 | **<0.001** |
|  | **PregnantY** | 0.133 | 0.013 | 10.07 | **<0.001** |
| *M.schreibersii* nursing/no-nursing females | (Intercept) | -1.245 | 0.005 | -237.040 | **<0.001** |
|  | **NipplesY** | 0.046 | 0.01 | 4.607 | **<0.001** |
| *M. schreibersii* without pregnant and nursing females | (Intercept) | -1.260 | 0.005 | -229.428 | **<0.001** |
|  | GenderM | -0.012 | 0.007 | -1.823 | n.s. |
|  | **AgeJUV** | -0.032 | 0.009 | -3.380 | **<0.001** |
