# Supplementary material for: Seasonal patterns and host traits as drivers of ectoparasite abundance in 3 bat species (Miniopterus schreibersii, Rhinolophus euryale, and Rhinolophus ferrumequinum) in the Central Balkans
Source: Curr Zool. 2025 Dec 22;72(5):692–702. doi: 10.1093/cz/zoaf079 (PMC13690573; doi:10.1093/cz/zoaf079)
Supplement: Supplementary file 1 [file zoaf079_supplementary_data.zip › Supplementary Material 3 R3.docx]

**Supplementary Material 3**

**Table S3.1** Results of GLMs to assess the effects of sex, age, season and host body condition index (BCI) on **total abundance of all ectoparasites** in *Rhinolphus ferumequinum*, *R. euryale* and *M. schreibersii:* and **total abundance of specific ectoparasite species** in *Rhinolphus euryale* (*Phthiridium biarticulatum*) and *Miniopterus schreibersii* (*Ixodes simplex, Nycteribia schmidlii, Spinturnix psi)* (JUV – juvenile host, M - male host, Season categories: Spring - April and May, Summer - July and August, Autumn - September and October; BCI – body condition index, E - estimate, SE – standard error, Z- value of Z statistics, *P* - value of the test; statistical significance: *P* < 0.05, n.s. – not statistically significant)

| **Ectoparasite** | **Variable** | **Estimate** | **Std. Error** | **Z value** | ***P*-value** |
| --- | --- | --- | --- | --- | --- |
| Ectoparasites Total in *R. ferrumequinum* | **(Intercept)** | **1.983** | **0.942** | **2.057** | **0.039** |
|  | **SexM** | **-1.177** | **0.316** | **-3.717** | **<0.001** |
|  | SeasonSpring | -0.210 | 0.364 | -0.577 | n.s. |
|  | SeasonSummer | -0.400 | 0.431 | -0.928 | n.s. |
|  | BCI | -2.579 | 2.171 | -1.188 | n.s. |
| Ectoparasites Total in *R. euryale* | **(Intercept)** | **3.045** | **1.272** | **2.394** | **0.016** |
|  | SexM | -0.303 | 0.280 | -1.084 | n.s. |
|  | **SeasonSpring** | **-0.805** | **0.385** | **-2.088** | **0.036** |
|  | SeasonSummer | 0.033 | 0.330 | 0.100 | n.s. |
|  | **BCI** | **-10.796** | **5.366** | **-2.012** | **0.044** |
| *P. biarticulatum* Total in *R. euryale* | **(Intercept)** | **3.9183** | **1.484** | **2.640** | **0.008** |
|  | SexM | -0.444 | 0.290 | -1.528 | n.s. |
|  | SeasonSpring | -0.290 | 0.418 | -0.694 | n.s. |
|  | **SeasonSummer** | **1.153** | **0.347** | **3.318** | **<0.001** |
|  | **BCI** | **-19.059** | **6.432** | **-2.963** | **<0.001** |
| Ectoparasites Total in *M. schreibersii* | **(Intercept)** | **0.561** | **0.281** | **1.995** | **0.046** |
|  | SexM | 0.169 | 0.093 | 1.815 | n.s. |
|  | AgeJUV | 0.009 | 0.202 | 0.048 | n.s. |
|  | **SeasonSpring** | **1.121** | **0.086** | **13.030** | **<0.001** |
|  | **SeasonSummer** | **1.064** | **0.085** | **12.408** | **<0.001** |
|  | **BCI** | **2.268** | **0.912** | **2.485** | **0.013** |
|  | SexM:AgeJUV | -0.184 | 0.265 | -0.693 | n.s. |
|  | **SexM:SeasonSpring** | **-0.717** | **0.130** | **-5.491** | **<0.001** |
|  | **SexM:SeasonSummer** | **-0.718** | **0.126** | **-5.695** | **<0.001** |
|  | AgeJUV:SeasonSummer | 0.299 | 0.233 | 1.284 | n.s. |
|  | SexM:AgeJUV:SeasonSummer | 0.462 | 0.317 | 1.456 | n.s. |
| *I.simplex* Total in *M. schreibersii* | **Intercept** | **-2.017** | **0.276** | **-7.287** | **< 0.001** |
|  | AgeJUV | 0.301 | 0.482 | 0.624 | n.s. |
|  | **SeasonSpring** | **1.809** | **0.217** | **8.312** | **< 0.001** |
|  | **SeasonSummer** | **1.712** | **0.214** | **7.968** | **< 0.001** |
|  | **SexM** | **-0.453** | **0.143** | **-3.166** | **0.001** |
|  | BCI | 0.002 | 0.001 | 1.811 | n.s. |
|  | AgeJUV:SeasonSummer | 0.510 | 0.530 | 0.962 | n.s. |
| *N. schmidlii* Total in *M. schreibersii* | (Intercept) | -0.472 | 0.359 | -1.316 | n.s. |
|  | SexM | 0.166 | 0.105 | 1.578 | n.s. |
|  | SeasonSpring | -0.176 | 0.122 | -1.575 | n.s. |
|  | **SeasonSummer** | **0.690** | **0.098** | **7.040** | **<0.001** |
|  | **AgeJUV** | **-0.356** | **0.099** | **-3.575** | **<0.001** |
|  | **BCI** | **4.787** | **1.178** | **4.063** | **<0.001** |
|  | SexM:SeasonSpring | -0.166 | 0.169 | -0.981 | n.s. |
|  | **SexM:SeasonSummer** | **-0.507** | **0.142** | **-3.575** | **<0.001** |
| *S. psi* Total in *M. schreibersii* | (Intercept) | -0.738 | 0.564 | -1.310 | n.s. |
|  | SexM | 0.312 | 0.216 | 1.442 | n.s. |
|  | **SeasonSpring** | **2.999** | **0.192** | **15.569** | **<0.001** |
|  | **SeasonSummer** | **2.220** | **0.185** | **11.972** | **<0.001** |
|  | **AgeJUV** | **1.104** | **0.141** | **7.821** | **<0.001** |
|  | BCI | -1.497 | 1.847 | -0.811 | n.s. |
|  | **SexM:SeasonSpring** | **-1.178** | **0.275** | **-4.270** | **<0.001** |
|  | **SexM:SeasonSummer** | **-1.030** | **0.260** | **-3.960** | **<0.001** |

**Table S3.2** Results of GLMs to assess the **effects of pregnancy in female hosts** in spring season on the total abundance of ectoparasites and total abundance of individual ectoparasite species: *Ixodes simplex*, *Nycteribia schmidlii* and *Spinturnix psi* in *Miniopterus schreibersii* (Pregnant Y – pregnant female host, E - estimate, SE – standard error, Z- value of Z statistics, *P -* value of the test; statistical significance: *P* < 0.05, n.s. – not statistically significant).

| **Ectoparasite** | **Variable** | **Estimate** | **Std. Error** | **Z value** | ***P*** **-value** |
| --- | --- | --- | --- | --- | --- |
| Ectoparasites Total | (Intercept) | 2.197 | 0.081 | 26.892 | <0.001 |
|  | **PregnantY** | **0.343** | **0.126** | **2.724** | **0.006** |
| *I.simplex* Total | (Intercept) | -0.189 | 0.203 | -0.932 | n.s. |
|  | **PregnantY** | **0.775** | **0.300** | **2.584** | **0.009** |
| *N. schmidlii* Total | (Intercept) | 0.384 | 0.134 | 2.871 | 0.004 |
|  | **PregnantY** | **0.784** | **0.191** | **4.041** | **<0.001** |
| *S. psi* Total | (Intercept) | 1.779 | 0.121 | 14.628 | <0.001 |
|  | PregnantY | 0.076 | 0.190 | 0.404 | n.s. |

**Table S3.3** Results of GLMs to assess the **effects of nursing in female hosts** in summer season on the total abundance of ectoparasites and total abundance of individual ectoparasite species: *Ixodes simplex*, *Nycteribia schmidlii* and *Spinturnix psi* in *Miniopterus schreibersii* (Nipples Y – female host who has breastfed, E - estimate, SE – standard error, Z- value of Z statistics, *P* - value of the test; statistical significance: *P* < 0.05).

| **Ectoparasite** | **Variable** | **Estimate** | **Std. Error** | **Z value** | ***P*** **-value** |
| --- | --- | --- | --- | --- | --- |
| Ectoparasites Total | (Intercept) | 2.187 | 0.064 | 33.961 | <0.001 |
|  | NipplesY | 0.184 | 0.099 | 1.855 | n.s. |
| *I.simplex* Total | (Intercept) | -0.488 | 0.186 | -2.620 | 0.008 |
|  | **NipplesY** | **1.038** | **0.261** | **3.978** | **<0.001** |
| *N. schmidlii* Total | (Intercept) | 1.562 | 0.089 | 17.494 | <0.001 |
|  | NipplesY | 0.101 | 0.139 | 0.731 | n.s. |
| *S. psi* Total | (Intercept) | 1.087 | 0.129 | 8.365 | <0.001 |
|  | NipplesY | 0.000 | 0.204 | 0.001 | n.s. |

**Table S3.4** Results of Tukey's post hoc tests - effect of sex, age and season on the abundance of ectoparasites on *Miniopterus schreibersii* individuals (JUV – juvenile *M. schreibersii*, AD – adult *M. schreibersii*, F –female *M. schreibersii*, M - male *M. schreibersii*, Season categories: Spring - April and May, Summer - July and August, Autumn - September and October; E - estimate, SE – standard error), Z- value of Z statistics, *P -* value of the test; statistical significance: *P* < 0.05).

**Table S3.4a** Tukey’s HSD test for seasonal, age and sex-related differences in the total abundance of ectoparasites in *Miniopterus schreibersii*.

| **Contrast** | **Estimate** | **SE** | **Z Ratio** | ***P*** *-*  **value** |
| --- | --- | --- | --- | --- |
| F AD Autumn - M AD Autumn | -0.16999 | 0.0937 | -1.815 | 0.7262 |
| F AD Autumn - F JUV Autumn | -0.00980 | 0.2030 | -0.048 | 1.0000 |
| F AD Autumn - M JUV Autumn | 0.00433 | 0.1770 | 0.024 | 1.0000 |
| F AD Autumn - F AD Spring | -1.12132 | 0.0861 | -13.030 | <.0001 |
| F AD Autumn - M AD Spring | -0.57369 | 0.1010 | -5.653 | <.0001 |
| F AD Autumn - F AD Summer | -1.06473 | 0.0858 | -12.408 | <.0001 |
| F AD Autumn - M AD Summer | -0.51609 | 0.0940 | -5.492 | <.0001 |
| F AD Autumn - F JUV Summer | -1.37404 | 0.1250 | -10.952 | <.0001 |
| F AD Autumn - M JUV Summer | -1.10410 | 0.1300 | -8.461 | <.0001 |
| M AD Autumn - F JUV Autumn | 0.16019 | 0.2020 | 0.795 | 0.9987 |
| M AD Autumn - M JUV Autumn | 0.17432 | 0.1760 | 0.989 | 0.9929 |
| M AD Autumn - F AD Spring | -0.95133 | 0.0865 | -10.997 | <.0001 |
| M AD Autumn - M AD Spring | -0.40370 | 0.0996 | -4.055 | 0.0020 |
| M AD Autumn - F AD Summer | -0.89474 | 0.0855 | -10.467 | <.0001 |
| M AD Autumn - M AD Summer | -0.34610 | 0.0935 | -3.700 | 0.0082 |
| M AD Autumn - F JUV Summer | -1.20405 | 0.1250 | -9.648 | <.0001 |
| M AD Autumn - M JUV Summer | -0.93411 | 0.1300 | -7.189 | <.0001 |
| F JUV Autumn - M JUV Autumn | 0.01413 | 0.2480 | 0.057 | 1.0000 |
| F JUV Autumn - F AD Spring | -1.11152 | 0.1990 | -5.591 | <.0001 |
| F JUV Autumn - M AD Spring | -0.56390 | 0.2000 | -2.819 | 0.1298 |
| F JUV Autumn - F AD Summer | -1.05493 | 0.1970 | -5.357 | <.0001 |
| F JUV Autumn - M AD Summer | -0.50629 | 0.2000 | -2.527 | 0.2537 |
| F JUV Autumn - F JUV Summer | -1.36424 | 0.2160 | -6.320 | <.0001 |
| F JUV Autumn - M JUV Summer | -1.09430 | 0.2190 | -4.995 | <.0001 |
| M JUV Autumn - F AD Spring | -1.12565 | 0.1730 | -6.516 | <.0001 |
| M JUV Autumn - M AD Spring | -0.57802 | 0.1760 | -3.280 | 0.0349 |
| M JUV Autumn - F AD Summer | -1.06906 | 0.1710 | -6.244 | <.0001 |
| M JUV Autumn - M AD Summer | -0.52041 | 0.1750 | -2.970 | 0.0873 |
| M JUV Autumn - F JUV Summer | -1.37837 | 0.1930 | -7.137 | <.0001 |
| M JUV Autumn - M JUV Summer | -1.10843 | 0.1970 | -5.638 | <.0001 |
| F AD Spring - M AD Spring | 0.54763 | 0.0936 | 5.848 | <.0001 |
| F AD Spring - F AD Summer | 0.05659 | 0.0776 | 0.729 | 0.9993 |
| F AD Spring - M AD Summer | 0.60524 | 0.0865 | 7.000 | <.0001 |
| F AD Spring - F JUV Summer | -0.25272 | 0.1200 | -2.111 | 0.5199 |
| F AD Spring - M JUV Summer | 0.01722 | 0.1250 | 0.138 | 1.0000 |
| M AD Spring - F AD Summer | -0.49104 | 0.0900 | -5.458 | <.0001 |
| M AD Spring - M AD Summer | 0.05761 | 0.0972 | 0.592 | 0.9999 |
| M AD Spring - F JUV Summer | -0.80034 | 0.1260 | -6.336 | <.0001 |
| M AD Spring - M JUV Summer | -0.53041 | 0.1320 | -4.028 | 0.0023 |
| F AD Summer - M AD Summer | 0.54865 | 0.0847 | 6.479 | <.0001 |
| F AD Summer - F JUV Summer | -0.30930 | 0.1180 | -2.621 | 0.2077 |
| F AD Summer - M JUV Summer | -0.03937 | 0.1240 | -0.319 | 1.0000 |
| M AD Summer - F JUV Summer | -0.85795 | 0.1240 | -6.929 | <.0001 |
| M AD Summer - M JUV Summer | -0.58802 | 0.1290 | -4.556 | 0.0002 |
| F JUV Summer - M JUV Summer | 0.26993 | 0.1530 | 1.766 | 0.7565 |

**Table S3.4b** Tukey’s HSD test for seasonal and age-related differences in total abundance of *Ixodes simplex* in *Miniopterus schreibersii*.

| **Contrast** | **Estimate** | **SE** | **Z Ratio** | ***P****-*  **value** |
| --- | --- | --- | --- | --- |
| AD Autumn - JUV Autumn | -0.2602 | 0.481 | -0.541 | 0.9831 |
| AD Autumn - AD Spring | -1.7946 | 0.217 | -8.277 | <.0001 |
| AD Autumn - AD Summer | -1.6967 | 0.214 | -7.936 | <.0001 |
| AD Autumn - JUV Summer | -2.4874 | 0.269 | -9.231 | <.0001 |
| JUV Autumn - AD Spring | -1.5344 | 0.456 | -3.363 | 0.0069 |
| JUV Autumn - AD Summer | -1.4365 | 0.456 | -3.151 | 0.0141 |
| JUV Autumn - JUV Summer | -2.2272 | 0.482 | -4.624 | <.0001 |
| AD Spring - AD Summer | 0.0979 | 0.162 | 0.604 | 0.9745 |
| AD Spring - JUV Summer | -0.6928 | 0.228 | -0.541 | 0.0201 |
| AD Summer - JUV Summer | -0.7907 | 0.226 | -8.277 | 0.0044 |

**Table S3.4c** Tukey’s HSD test for seasonal and sex-related differences in the total abundance of *Nycteribia schmidlii* in *Miniopterus schreibersii*.

| **Contrast** | **Estimate** | **SE** | **Z Ratio** | ***P****-*  **value** |
| --- | --- | --- | --- | --- |
| F Autumn - M Autumn | -0.166317 | 0.1050 | -1.578 | 0.6131 |
| F Autumn - F Spring | 0.176833 | 0.1120 | 1.575 | 0.6150 |
| F Autumn - M Spring | 0.177115 | 0.1310 | 1.351 | 0.7560 |
| F Autumn - F Summer | -0.690172 | 0.0980 | -7.040 | <.0001 |
| F Autumn - M Summer | -0.348661 | 0.1060 | -3.301 | 0.0124 |
| M Autumn - F Spring | 0.343150 | 0.1120 | 3.051 | 0.0277 |
| M Autumn - M Spring | 0.343432 | 0.1290 | 2.668 | 0.0817 |
| M Autumn - F Summer | -0.523854 | 0.0966 | -5.423 | <.0001 |
| M Autumn - M Summer | -0.182344 | 0.1040 | -1.751 | 0.4977 |
| F Spring - M Spring | 0.000282 | 0.1360 | 0.002 | 1.0000 |
| F Spring - F Summer | -0.867004 | 0.1060 | -8.187 | <.0001 |
| F Spring - M Summer | -0.525493 | 0.1130 | -4.645 | <.0001 |
| M Spring - F Summer | -0.867286 | 0.1210 | -7.148 | <.0001 |
| M Spring - M Summer | -0.525776 | 0.1270 | -4.126 | 0.0005 |
| F Summer - M Summer | 0.341511 | 0.0954 | 3.579 | 0.0047 |

**Table S3.4d** Tukey’s HSD test for seasonal and sex-related differences in the total abundance of *Spinturnix psi* in *Miniopterus schreibersii*.

| **Contrast** | **Estimate** | **SE** | **Z Ratio** | ***P*** *-* **value** |
| --- | --- | --- | --- | --- |
| F Autumn - M Autumn | -0.312 | 0.217 | -1.442 | 0.7012 |
| F Autumn - F Spring | -3.000 | 0.193 | -15.569 | <.0001 |
| F Autumn - M Spring | -2.134 | 0.213 | -9.996 | <.0001 |
| F Autumn - F Summer | -2.221 | 0.186 | -11.972 | <.0001 |
| F Autumn - M Summer | -1.503 | 0.196 | -7.675 | <.0001 |
| M Autumn - F Spring | -2.687 | 0.182 | -14.772 | <.0001 |
| M Autumn - M Spring | -1.821 | 0.201 | -9.040 | <.0001 |
| M Autumn - F Summer | -1.908 | 0.172 | -11.068 | <.0001 |
| M Autumn - M Summer | -1.190 | 0.183 | -6.501 | <.0001 |
| F Spring - M Spring | 0.866 | 0.174 | 4.986 | <.0001 |
| F Spring - F Summer | 0.779 | 0.143 | 5.457 | <.0001 |
| F Spring - M Summer | 1.497 | 0.157 | 9.520 | <.0001 |
| M Spring - F Summer | -0.087 | 0.165 | -0.526 | 0.9951 |
| M Spring - M Summer | 0.631 | 0.177 | 3.557 | 0.0050 |
| F Summer - M Summer | 0.718 | 0.145 | 4.964 | <.0001 |
