# Supplementary material for: Seasonal patterns and host traits as drivers of ectoparasite abundance in 3 bat species (Miniopterus schreibersii, Rhinolophus euryale, and Rhinolophus ferrumequinum) in the Central Balkans
Source: Curr Zool. 2025 Dec 22;72(5):692–702. doi: 10.1093/cz/zoaf079 (PMC13690573; doi:10.1093/cz/zoaf079)
Supplement: Supplementary file 1 [file zoaf079_supplementary_data.zip › Supplementary Material 4 R3.docx]

**Supplementary Material 4**

**Table S4.1** Results of GLMs to assess the **effects of sex, age, season and host body condition index (BCI) on life stages indicative of reproduction of ectoparasites in Miniopterus schreibersii:** *Ixodes simplex, Nycteribia schmidlii* and *Spinturnix psi* (JUV – juvenile host, M - male host, Season categories: Spring - April and May, Summer - July and August, Autumn - September and October; BCI – body condition index, E - estimate, SE – standard error, Z- value of Z statistics, *P* - value of the test; statistical significance: *P* < 0.05, n.s. – not statistically significant).

| **Ectoparasite** | **Variable** | **Estimate** | **Std. Error** | **Z value** | ***P*-value** |
| --- | --- | --- | --- | --- | --- |
| *I.simplex* females | **(Intercept)** | -5.516 | 0.845 | -6.528 | **<0.001** |
|  | SexM | -0.145 | 0.349 | -0.416 | n.s. |
|  | **AgeJUV** | 2.417 | 0.381 | 6.337 | **<0.001** |
|  | **SeasonSpring** | 2.007 | 0.881 | 2.278 | **0.022** |
|  | **SeasonSummer** | 2.930 | 0.737 | 3.973 | **<0.001** |
|  | BCI | -0.002 | 0.002 | -0.35 | n.s. |
| *I.simplex* nymphs | **(Intercept)** | -3.031 | 0.327 | -9.255 | **< 0.001** |
|  | SexM | -0.254 | 0.167 | -1.525 | n.s. |
|  | AgeJUV | 0.377 | 0.245 | 1.534 | n.s. |
|  | **SeasonSpring** | 1.997 | 0.258 | 7.715 | **< 0.001** |
|  | **SeasonSummer** | 1.945 | 0.246 | 7.902 | **< 0.001** |
|  | **BCI** | 0.003 | 0.001 | 2.940 | **0.003** |
| *I.simplex* larvae | (Intercept) | -1.353 | 1.212 | -1.116 | n.s. |
|  | SexM | 0.016 | 0.493 | 0.034 | n.s. |
|  | **SeasonSpring** | 2.193 | 0.401 | 5.459 | **< 0.001** |
|  | **SeasonSummer** | 1.743 | 0.393 | 4.430 | **< 0.001** |
|  | AgeJUV | 0.169 | 0.317 | 0.535 | n.s. |
|  | BCI | -4.954 | 3.980 | -1.245 | n.s. |
|  | **SexM:SeasonSpring** | -1.661 | 0.633 | -2.622 | **0.008** |
|  | **SexM:SeasonSummer** | -1.041 | 0.587 | -1.744 | n.s. |
| *N. schmidlii* pregnant females | (Intercept) | -2.157 | 0.340 | -6.344 | **< 0.001** |
|  | AgeJUV | -0.273 | 0.242 | -1.31 | n.s. |
|  | BCI | 0.000 | 0.000 | 0.871 | n.s. |
|  | **SexM** | 1.250 | 0.330 | 3.786 | **< 0.001** |
|  | **SeasonSpring** | 2.332 | 0.313 | 7.429 | **< 0.001** |
|  | **SeasonSummer** | 1.693 | 0.311 | 5.428 | **< 0.001** |
|  | **SexM:SeasonSpring** | -1.276 | 0.392 | -3251 | **0.001** |
|  | **SexM:SeasonSummer** | -1.665 | 0.387 | -4.294 | **< 0.001** |
| *S. psi* pregnant females | (Intercept) | -3.963 | 0.951 | -4.166 | **< 0.001** |
|  | **SexM** | -0.643 | 0.208 | -3.093 | **0.001** |
|  | **AgeJUV** | 1.016 | 0.259 | 3.915 | **< 0.001** |
|  | **SeasonSpring** | 1.396 | 0.431 | 3.232 | **0.001** |
|  | SeasonSummer | 0.727 | 0.421 | 1.729 | n.s. |
|  | **BCI** | 7.883 | 2.970 | 2.654 | **0.007** |
| *S. psi* pre-adults | (Intercept) | -5.772 | 1.136 | -5.078 | **< 0.001** |
|  | SexM | 1.284 | 0.822 | 1.562 | n.s. |
|  | **SeasonSpring** | 5.215 | 0.743 | 7.017 | **< 0.001** |
|  | **SeasonSummer** | 3.621 | 0.740 | 4.891 | **< 0.001** |
|  | **AgeJUV** | 1.762 | 0.239 | 7.351 | **< 0.001** |
|  | BCI | 3.378 | 3.020 | 1.119 | n.s. |
|  | **SexM:SeasonSpring** | -2.187 | 0.860 | -2.543 | **0.011** |
|  | **SexM:SeasonSummer** | -1.944 | 0.859 | -2.261 | **0.023** |

**Table S4.2** Results of GLMs to assess the **effects of pregnancy in female hosts in spring season on life stages indicative of reproduction of ectoparasites in *Miniopterus schreibersii*:** *Ixodes. simplex* nymphs, *I. simplex* larvae, *Nycteribia schmidlii* pregnant females, *Spinturnix psi* pregnant females *and S.psi* pre-adults in *M. schreibersii* (Pregnant Y – pregnant female host, E - estimate, SE – standard error, Z- value of Z statistics, *P* - value of the test; statistical significance: *P* < 0.05, n.s. – not statistically significant).

| **Ectoparasite** | **Variable** | **Estimate** | **Std. Error** | **Z value** | ***P* -value** |
| --- | --- | --- | --- | --- | --- |
| *I. simplex* nymphs | (Intercept) | -0.960 | 0.252 | -3.808 | <0.001 |
|  | **PregnantY** | **1.047** | **0.355** | **2.946** | **<0.001** |
| *I.simplex* larvae | (Intercept) | -0.839 | 0.280 | -2.989 | 0.002 |
|  | PregnantY | 0.442 | 0.424 | 1.044 | n.s. |
| *N. schmidlii* pregnant females | (Intercept) | 0.126 | 0.169 | 0.746 | n.s. |
|  | PregnantY | 0.395 | 0.226 | 1.749 | n.s. |
| *S. psi* pregnant females | (Intercept) | -0.287 | 0.173 | -1.662 | n.s. |
|  | PregnantY | 0.200 | 0.270 | 0.742 | n.s. |
| *S. psi* pre-adults | (Intercept) | 0.393 | 0.165 | 2.373 | 0.017 |
|  | PregnantY | 0.088 | 0.258 | 0.341 | n.s. |

**Table S4.3** Results of GLMs to assess the **effects of nursing in female hosts in summer season on life stages indicative of reproduction of ectoparasites in *Miniopterus schreibersii****: Ixodes. simplex* nymphs, *I. simplex* larvae, *Nycteribia schmidlii* pregnant females, *Spinturnix psi* pregnant females *and S.psi* pre-adults in *M. schreibersii* (Nipples Y – a female host who has breastfed, E - estimate, SE – standard error, Z- value of Z statistics, *P* - value of the test; statistical significance: *P* < 0.05, n.s. – not statistically significant).

| **Ectoparasite** | **Variable** | **Estimate** | **Std. Error** | **Z value** | ***P*-value** |
| --- | --- | --- | --- | --- | --- |
| *I. simplex* nymphs | (Intercept) | -1.110 | 0.221 | -5.023 | <0.001 |
|  | **NipplesY** | **1.142** | **0.293** | **3.897** | **<0.001** |
| *I.simplex* larvae | (Intercept) | -1.432 | 0.269 | -5.318 | <0.001 |
|  | **NipplesY** | **0.976** | **0.368** | **2.651** | **0.008** |
| *N. schmidlii* pregnant females | (Intercept) | 0.015 | 0.161 | 0.093 | n.s. |
|  | **NipplesY** | **-1.002** | **0.304** | **-3.289** | **0.001** |
| *S. psi* pregnant females | (Intercept) | -0.896 | 0.237 | -3.772 | <0.001 |
|  | NipplesY | -0.202 | 0.408 | -0.496 | n.s. |
| *S. psi* pre-adults | (Intercept) | -1.110 | 0.276 | -4.021 | <0.001 |
|  | NipplesY | -0.211 | 0.447 | -0.473 | n.s. |

**Table S4.4** Results of Tukey's post hoc tests - effect of sex, age and season on life stages indicative of reproduction of ectoparasites in *Miniopterus schreibersii* ( F –female *M. schreibersii*, M - male *M. schreibersii*, Season categories: Spring - April and May, Summer - July and August, Autumn - September and October; E - estimate, SE – standard error), Z- value of Z statistics, *P* - value of the test; statistical significance: *P* < 0.05).

**Table S4.4a**: Tukey’s HSD test for seasonal and sex-related differences in abundance r of *Ixodes simplex* larvae in *Miniopterus schreibersii*.

| **Contrast** | **Estimate** | **SE** | **Z Ratio** | ***P* - value** |
| --- | --- | --- | --- | --- |
| F Autumn - M Autumn | -0.0167 | 0.493 | -0.034 | 1.0000 |
| F Autumn - F Spring | -2.1932 | 0.402 | -5.459 | <.0001 |
| F Autumn - M Spring | -0.5480 | 0.499 | -1.098 | 0.8822 |
| F Autumn - F Summer | -1.7439 | 0.394 | -4.430 | 0.0001 |
| F Autumn - M Summer | -0.7190 | 0.441 | -1.632 | 0.5771 |
| M Autumn - F Spring | -2.1766 | 0.403 | -5.399 | <.0001 |
| M Autumn - M Spring | -0.5314 | 0.495 | -1.074 | 0.8917 |
| M Autumn - F Summer | -1.7272 | 0.391 | -4.415 | 0.0001 |
| M Autumn - M Summer | -0.7023 | 0.438 | -1.605 | 0.5955 |
| F Spring - M Spring | 1.6452 | 0.405 | 4.061 | 0.0007 |
| F Spring - F Summer | 0.4494 | 0.274 | 1.639 | 0.5727 |
| F Spring - M Summer | 1.4743 | 0.340 | 4.338 | 0.0002 |
| M Spring - F Summer | -1.1959 | 0.394 | -3.038 | 0.0288 |
| M Spring - M Summer | -0.1710 | 0.440 | -0.389 | 0.9989 |
| F Summer - M Summer | 1.0249 | 0.320 | 3.203 | 0.0171 |

**Table S4.4b**: Tukey’s HSD test for seasonal and sex-related differences in abundance of *Nycteribia schmidlii* pregnant females in *Miniopterus schreibersii*.

| **Contrast** | **Estimate** | **SE** | **Z Ratio** | ***P* - value** |
| --- | --- | --- | --- | --- |
| F Autumn - M Autumn | -1.2509 | 0.330 | -3.786 | 0.0021 |
| F Autumn - F Spring | -2.3324 | 0.314 | -7.429 | <.0001 |
| F Autumn - M Spring | -2.3069 | 0.337 | -6.842 | <.0001 |
| F Autumn - F Summer | -1.6933 | 0.312 | -5.428 | <.0001 |
| F Autumn - M Summer | -1.2791 | 0.334 | -3.825 | 0.0018 |
| M Autumn - F Spring | -1.0814 | 0.210 | -5.138 | <.0001 |
| M Autumn - M Spring | -1.0559 | 0.236 | -4.472 | 0.0001 |
| M Autumn - F Summer | -0.4424 | 0.203 | -2.181 | 0.2465 |
| M Autumn - M Summer | -0.0282 | 0.235 | -0.120 | 1.0000 |
| F Spring - M Spring | 0.0255 | 0.221 | 0.116 | 1.0000 |
| F Spring - F Summer | 0.6390 | 0.181 | 3.524 | 0.0057 |
| F Spring - M Summer | 1.0532 | 0.218 | 4.822 | <.0001 |
| M Spring - F Summer | 0.6135 | 0.203 | 3.021 | 0.0303 |
| M Spring - M Summer | 1.0277 | 0.235 | 4.376 | 0.0002 |
| F Summer - M Summer | 0.4142 | 0.204 | 2.033 | 0.3237 |

**Table S4.4c**: Tukey’s HSD test for seasonal and sex-related differences in the abundance of *Spinturnix psi* pre-adults in *Miniopterus schreibersii*.

| **Contrast** | **Estimate** | **SE** | **Z Ratio** | ***P* - Value** |
| --- | --- | --- | --- | --- |
| F Autumn - M Autumn | -1.284 | 0.822 | -1.562 | 0.6234 |
| F Autumn - F Spring | -5.215 | 0.743 | -7.017 | <.0001 |
| F Autumn - M Spring | -4.312 | 0.758 | -5.686 | <.0001 |
| F Autumn - F Summer | -3.622 | 0.741 | -4.891 | <.0001 |
| F Autumn - M Summer | -2.962 | 0.752 | -3.941 | 0.0011 |
| M Autumn - F Spring | -3.931 | 0.427 | -9.214 | <.0001 |
| M Autumn - M Spring | -3.027 | 0.451 | -6.705 | <.0001 |
| M Autumn - F Summer | -2.337 | 0.419 | -5.585 | <.0001 |
| M Autumn - M Summer | -1.678 | 0.437 | -3.841 | 0.0017 |
| F Spring - M Spring | 0.903 | 0.256 | 3.531 | 0.0055 |
| F Spring - F Summer | 1.593 | 0.224 | 7.120 | <.0001 |
| F Spring - M Summer | 2.253 | 0.267 | 8.446 | <.0001 |
| M Spring - F Summer | 0.690 | 0.267 | 2.581 | 0.1020 |
| M Spring - M Summer | 1.350 | 0.304 | 4.435 | 0.0001 |
| F Summer - M Summer | 0.660 | 0.254 | 2.600 | 0.0971 |
