# Supplementary material for: Seasonal patterns and host traits as drivers of ectoparasite abundance in 3 bat species (Miniopterus schreibersii, Rhinolophus euryale, and Rhinolophus ferrumequinum) in the Central Balkans
Source: Curr Zool. 2025 Dec 22;72(5):692–702. doi: 10.1093/cz/zoaf079 (PMC13690573; doi:10.1093/cz/zoaf079)
Supplement: Supplementary file 1 [file zoaf079_supplementary_data.zip › Supplementary_Material_2_R3.docx]

**Supplementary Material 2**

An overview of the ectoparasite taxa (ticks, flies, and mites) identified on the three bat species (*Rhinolophus ferrumequinum, Rhinolophus euryale, Miniopterus schreibersii*), along with their respective abundances, is presented in Table S2.1.

**Table S2.1** Total abundance of parasite individuals recorded per host species.

| **Host bat species** | **Ectoparasites (ticks, flies, mites)** | **Total Number of Ectoparasites** |
| --- | --- | --- |
| *Rhinolophus ferrumequinum* | *Ixodes simplex* | 2 |
|  | *Ixodes vespertilionis* | 27 |
|  | *Nycteribia schmidlii* | 1 |
|  | ***Phthiridium biarticulatum*** | 78 |
|  | *Spinturnix psi* | 3 |
|  | *Spinturnix myoti* | 1 |
|  | *Eyndhovenia euryllis* | 49 |
|  | *Macronyssus granulossus* | 1 |
|  | *Rhinolophopsylla unipectinata* | 14 |
| *Rhinolophus euryale* | *Ixodes simplex* | 3 |
|  | *Ixodes vespertilionis* | 10 |
|  | *Nycteribia schmidlii* | 1 |
|  | *Nycteribia latrelii* | 1 |
|  | *Nycteribia pedicularia* | 1 |
|  | ***Phthiridium biarticulatum*** | 109 |
|  | *Penicillidia conspicua* | 2 |
|  | *Penicillidia dufour* | 1 |
|  | *Sprinturnix psi* | 8 |
|  | *Eyndhovenia euryllis* | 65 |
|  | *Rhinolophopsylla unipectinata* | 2 |
| *Miniopterus schreibersii* | ***Ixodes simplex*** | 603 |
|  | *Ixodes verpertilionis* | 5 |
|  | ***Nycteribia schmidlii*** | 2641 |
|  | *Nycteribia lateralii* | 3 |
|  | *Nycteribia vexata* | 3 |
|  | *Penicillidia conspicua* | 309 |
|  | *Penicillidia dufouri* | 80 |
|  | *Spinturnix spp.* | 54 |
|  | ***Spinturnix psi*** | 2306 |
|  | *Spinturnix myoti* | 41 |
|  | *Eyndhoevenia euryalis* | 1 |
|  | *Macronyssus granulossus* | 4 |
|  | *Macronyssus sp.* | 6 |

**** Bold parasites are the most numerous in a particular host.*

**Table S2.2** Sample size, prevalence, and the total abundance of ectoparasites in three analyzed species of bats (*Miniopterus schreibersii, Rhinolophus euryale*, and *Rhinolophus ferrumequinum*)

|  | Host gender | | Host age | | Host life cycle | | |
| --- | --- | --- | --- | --- | --- | --- | --- |
|  | Female | Male | Adult | Juvenile | Spring | Summer | Autumn |
| *M. scheibersii* | | | | | | | |
| Number of hosts | 480 | 409 | 777 | 112 | 241 | 335 | 313 |
| Prevalence % | 96.45 463/480 | 92.45 378/409 | 94.72  736/777 | 93.75  105/112 | 96.26  231/241 | 98.20  329/335 | 89.45  280/313 |
| Total number of ectoparasites | 3874 | 2182 | 5112 | 944 | 2018 | 2890 | 1148 |
| *R. euryale* | | | | | | | |
| Number of hosts | 73* | 81 | 144* | 10 | 30 | 51 | 75 |
| Prevalence % | 56.16  41/73 | 38.27  31/81 | 46.27  67/144 | 50  5/10 | 40  12/30 | 45.09  23/51 | 49.33  37/75 |
| Total number of ectoparasites | 111 | 92 | 172 | 31 | 20 | 61 | 122 |
| *R. ferrumequinum* | | | | | | | |
| Number of hosts | 58* | 57 | 104* | 16 | 36 | 29 | 54 |
| Prevalence % | 55.17  32/58 | 40.35  23/57 | 49.04  51/104 | 50  8/16 | 58.33  21/36 | 17.24  5/29 | 53.70  29/54 |
| Total number of ectoparasites | 134 | 40 | 129 | 45 | 46 | 27 | 101 |

**- The number of host individuals by sex and age is slightly lower than the total number because there were individuals for which the sex and age (NA) were not determined. Such individuals were not included in the models.*

**Table S2.3** Summary of ectoparasite abundance in *Miniopterus schreibersii*, showing total counts per parasite taxon and their distribution across host sex (female, male), age class (adult, juvenile), and season (spring, summer, autumn).

**Table S2. 3a** *Ixodes simplex* total, females, nymphs and larvae distribution in *Miniopterus schreibersii*.

|  | **Host *Miniopterus schreibersii*** | | | | | | | |
| --- | --- | --- | --- | --- | --- | --- | --- | --- |
| **Parasite** | **Total Number** | **Female** | **Male** | **Adult** | **Juvenile** | **Spring** | **Summer** | **Autumn** |
| ***Ixodes simplex* Total** | 603 | 411 | 192 | 482 | 121 | 223 | 332 | 48 |
| ***Ixodes simplex* Females** | 66 | 35 | 31 | 19 | 47 | 5 | 59 | 2 |
| ***Ixodes simplex* Nymphs** | 342 | 225 | 117 | 291 | 51 | 133 | 183 | 26 |
| ***Ixodes simplex* larvae** | 195 | 151 | 44 | 172 | 23 | 85 | 90 | 20 |

**Table S2. 3b** *Nycteribia scmidlii* total, females, pregnant females, and males distribution in *Miniopterus schreibersii*.

|  | **Host *Miniopterus schreibersii*** | | | | | | | |
| --- | --- | --- | --- | --- | --- | --- | --- | --- |
| **Parasite** | **Total Number** | **Female** | **Male** | **Adult** | **Juvenile** | **Spring** | **Summer** | **Autumn** |
| ***Nycteribia schmidlii* Total** | 2641 | 1539 | 1102 | 2371 | 270 | 487 | 1320 | 834 |
| ***Nycteribia schmidlii* females*** | 1373 | 831 | 542 | 1233 | 140 | 284 | 686 | 403 |
| ***Nycteribia schmidlii* pregnant** | 370 | 212 | 158 | 346 | 24 | 170 | 140 | 60 |
| ***Nycteribia schmidlii* males** | 1268 | 708 | 560 | 1138 | 130 | 203 | 634 | 431 |

**female category includes pregnant individuals*

**Table S2. 3c** *Spinturnix psi* total, females, pregnant females, males and pre-adult forms distribution in *Miniopterus schreibersii*.

|  | **Host *Miniopterus schreibersii*** | | | | | | | |
| --- | --- | --- | --- | --- | --- | --- | --- | --- |
| **Parasite** | **Total Number** | **Female** | **Male** | **Adult** | **Juvenile** | **Spring** | **Summer** | **Autumn** |
| ***S. psi* Total** | 2306 | 1614 | 692 | 1808 | 498 | 1118 | 1044 | 144 |
| ***S. psi* females*** | 996 | 690 | 306 | 796 | 200 | 508 | 438 | 50 |
| ***S. psi* pregnant** | 171 | 132 | 39 | 135 | 36 | 95 | 69 | 7 |
| ***S. psi* males** | 867 | 599 | 268 | 675 | 192 | 342 | 440 | 85 |
| ***S. psi* pre-adults** | 443 | 325 | 118 | 337 | 106 | 268 | 166 | 9 |

**female category includes pregnant individuals*
